# Multi-omics analysis reveals the key role of STIL in Li-Fraumeni syndrome and osteosarcoma

**DOI:** 10.1038/s41698-026-01432-y

**Published:** 2026-04-20

**Authors:** Yu Qiao, Jincen Hao, Fahu Yuan, Anna Curto-Vilalta, Long Tang, Rüdiger von Eisenhart-Rothe, Florian Hinterwimmer

**Affiliations:** 1 Department of Orthopaedics and Sports Orthopaedics, School of Medicine and Health, TUM University Hospital, Technical University of Munich, Munich, Germany; 2 Health Science Center, Peking University, Beijing, China; 3 School of Medicine, Jianghan University, Wuhan, China; 4 Institute for AI and Informatics in Medicine, School of Medicine and Health, TUM University Hospital, Technical University of Munich, Munich, Germany; 5 Department of Neurosurgery, School of Medicine and Health, TUM University Hospital, Technical University of Munich, Munich, Germany

**Keywords:** Bone cancer, Biomarkers, Mathematics and computing

## Abstract

Li-Fraumeni syndrome (LFS), driven by germline TP53 mutations, confers a markedly elevated risk of osteosarcoma (OS), yet the mechanisms beyond TP53 remain insufficiently defined. By integrating multi-omics analyses and in vitro validation, we identify SCL-interrupting locus (STIL) as a pivotal hub linking LFS to OS progression. We reveal that STIL negatively regulates p53 protein stability in a manner independent of TP53 mutation status, indicating that STIL can promote tumorigenesis by dampening p53 pathway activity and stability. Importantly, STIL displays genetic-context–dependent oncogenicity: it supports stemness across OS models, but more strongly drives invasion and metastatic potential in TP53-mutant backgrounds. Specifically, STIL is highly expressed in a population of high-stemness malignant cells (Pro-OSCs), where it maintains stemness and promotes bone destruction by activating PTN–NCL and FN1–CD44 pathways, while simultaneously remodeling the immune microenvironment via MIF and APP signaling to evade immune surveillance. Additionally, WEE1 inhibitors may represent a targeted vulnerability in STIL-high OS. In summary, the relationship between TP53 and STIL is not a simple linear upstream-downstream cascade, but reflects a highly context-dependent regulatory dynamic. STIL exerts oncogenic effects by regulating p53 stability and driving a “stemness-invasive” phenotype in the context of TP53 mutations. This also provides novel biomarkers and intervention targets for precision therapy.

## Introduction

Li–Fraumeni syndrome (LFS) was first described in 1969 by Frederick Pei Li and Joseph Fraumeni Jr. It is a rare autosomal dominant hereditary disorder associated with germline pathogenic/likely pathogenic (P/LP) variants in the tumor suppressor gene TP53^1,2^. Individuals with LFS exhibit a markedly elevated lifetime risk of developing cancer, with approximately 50% of carriers diagnosed with some form of malignancy before the age of 40, and the cumulative lifetime cancer risk exceeds 90%^3^. Among the broad spectrum of LFS-associated malignancies, osteosarcoma (OS) represents a hallmark tumor type^4^. Epidemiological studies indicate that OS is the most common bone malignancy in LFS families, with carriers exhibiting a risk of developing OS that is hundreds of times higher than the general population^5^. A French study found that approximately 30% of children with LFS developed OS^6^. Notably, LFS exhibits age-related characteristics of cancer development, with LFS-associated OS often arising during the transition from childhood to adolescence, coinciding with the first peak incidence of bone tumors^7^.

OS itself is a highly aggressive mesenchymal tumor and remains the most common primary bone cancer in children and young adults^8^. Its global incidence is approximately 3–4 cases per million per year, accounting for less than 1% of all malignant tumors, suggesting a potential association with specific genetic susceptibility syndromes^9,10^. Despite continuous optimization of therapeutic strategies, including neoadjuvant chemotherapy, over the past decades, the overall prognosis of OS has not substantially improved due to its pronounced invasiveness and high recurrence rate^11^. Currently, studies have identified multiple germline mutations that play a key role in OS susceptibility, including LFS and retinoblastoma syndrome^12^. Notably, the molecular link between LFS and OS is anchored in TP53^13^. Somatic TP53 mutations are the most common genetic alteration in sporadic OS, accounting for approximately half of all cases, while germline TP53 mutations define LFS-associated OS^14,15^. Therefore, identifying key downstream effectors or co-conspirators that drive tumorigenesis in the context of TP53 mutation is critical for developing new precision therapies. While the genetic overlap is known, the specific molecular network that translates the LFS genotype (TP53 mutation) into the OS phenotype remains incompletely understood.

To bridge this gap, we employed a systematic strategy integrating multi-omics bioinformatics and in vitro experiments. With the rapid advancement of artificial intelligence and single-cell sequencing technologies, we can now dissect complex disease mechanisms at unprecedented resolution. In this study, we identified the SCL-interrupting locus (STIL) as a key factor involved in the potential mechanism underlying LFS-associated OS by leveraging these multi-dimensional analytical strategies. To overcome the “black box” nature of traditional machine learning, we introduce SHapley Additive exPlanations (SHAP) technology to visualize the decision-making process of predictive models^16^. Furthermore, we leveraged single-cell RNA sequencing (scRNA-seq) to trace the expression of STIL in malignant cell trajectories and its impact on the tumor microenvironment (TME)^17,18^.

**Fig. 1:**
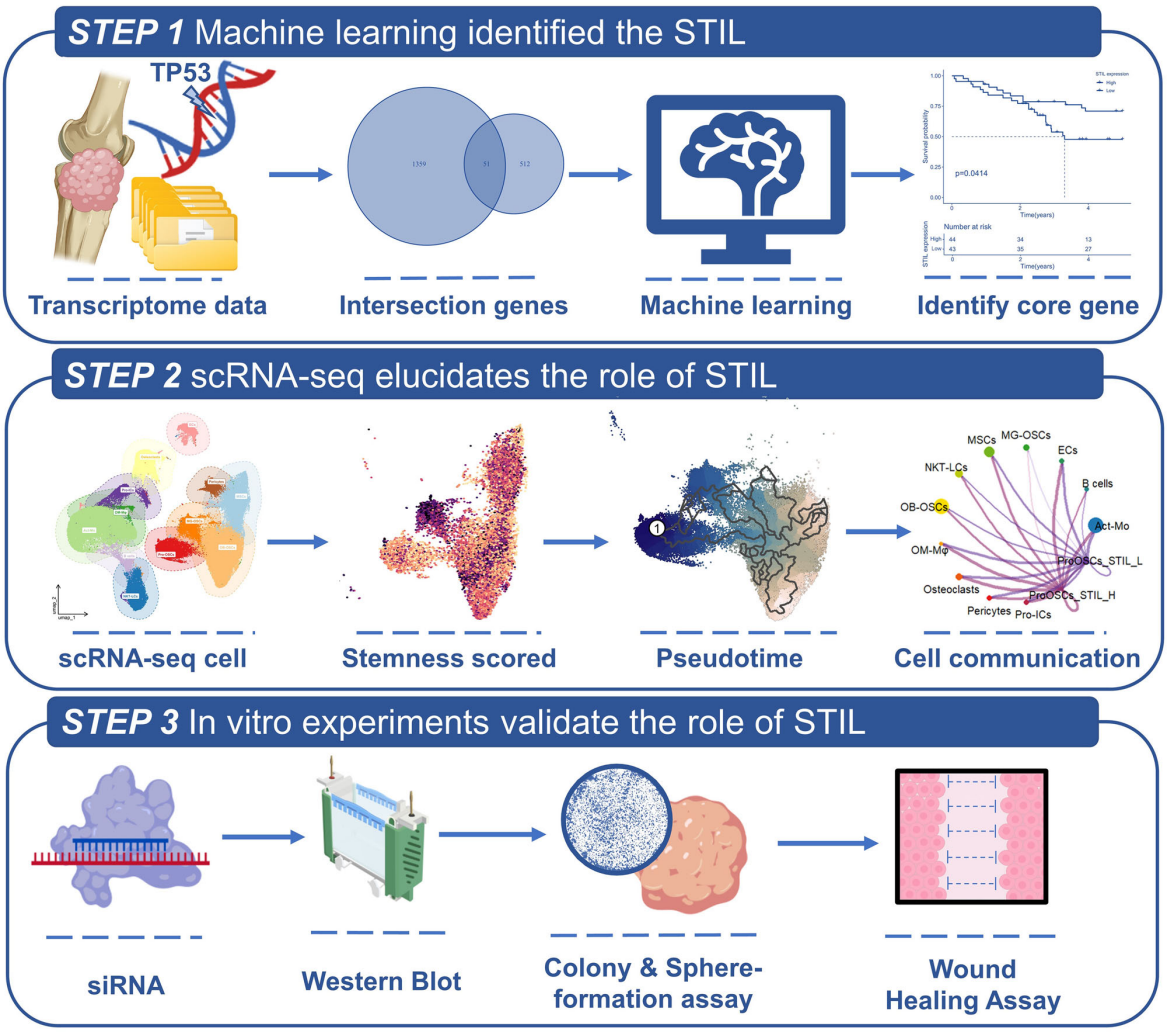
Schematic diagram of the study design for analyzing the key role of STIL in Li–Fraumeni syndrome and osteosarcoma patients.

Crucially, our experimental validation confirms that STIL serves not only as a biomarker but also as a functional factor, with its activity being context-dependent on the TP53 background. In the context of TP53 mutations, it is characterized by promoting cellular stemness and metastasis. By elucidating STIL’s role as a functional bridge between LFS and OS progression, this study aims to provide new insights into the pathogenesis of TP53 mutation-associated OS and establish STIL as a potential therapeutic target to overcome current clinical impasses. The workflow of this study is shown in Fig. 1.

## Results

### Identification of LFS-OS shared genes

**Fig. 2:**
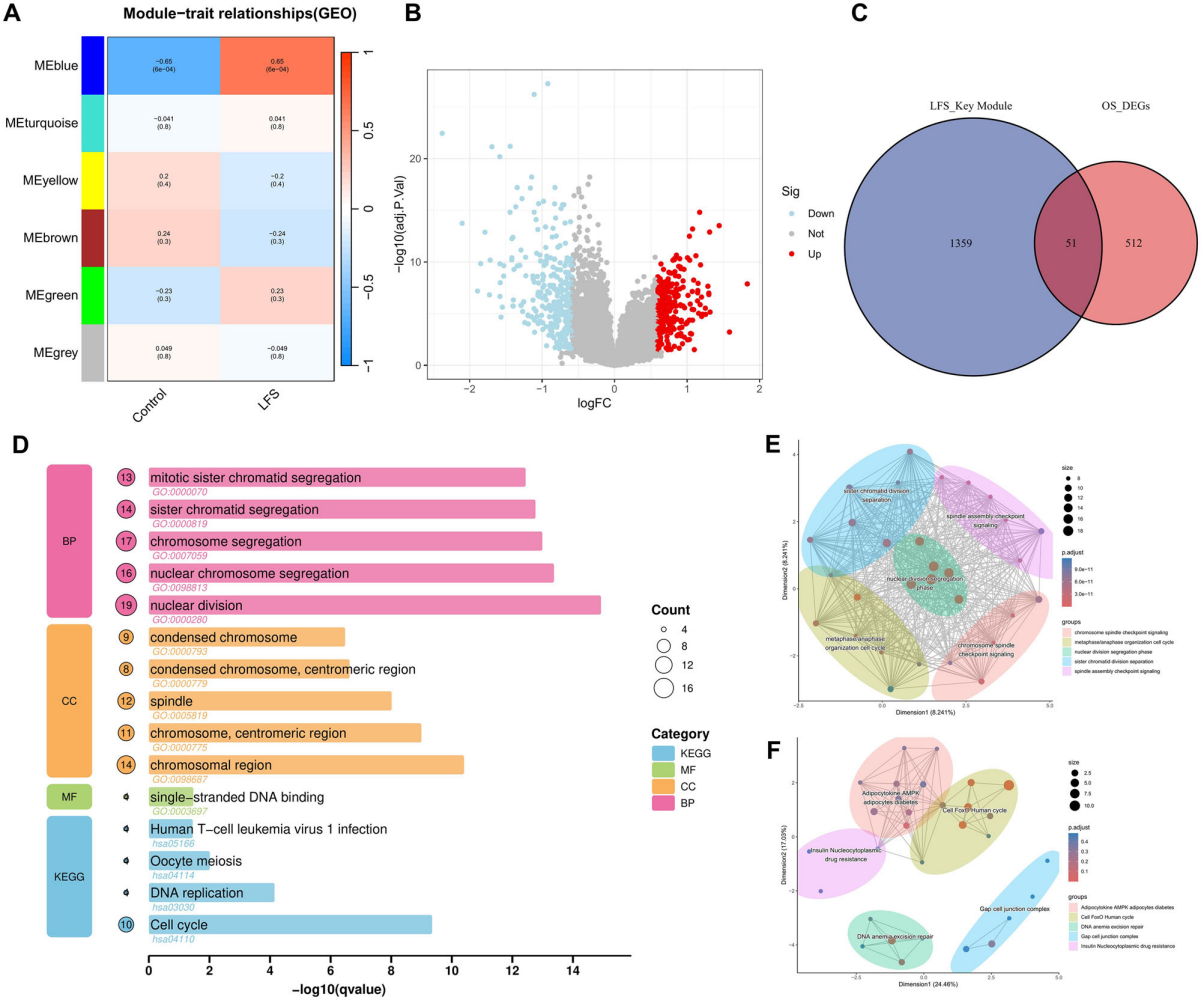
Integrated transcriptomic identification of key genes linking Li–Fraumeni Syndrome (LFS) and Osteosarcoma (OS). **A** Weighted gene co-expression network analysis of the LFS cohort identified six modules. The blue module showed the strongest correlation with the LFS phenotype (*r* = 0.65, *p* < 0.001). **B** Volcano plot of differentially expressed genes between osteosarcoma and control samples. Upregulated genes are marked in red, while downregulated genes are marked in blue. **C** The venn diagram identified 51 LFS-OS shared genes. **D** Enrichment analysis of biological processes, cellular components, molecular functions, and KEGG pathways for 51 intersecting genes. **E**, **F** Dimensionality reduction and clustering analysis of gene ontology and KEGG terms. The clustering network diagram reveals strong associations between cell cycle-related pathways and mitotic checkpoint signaling.

We identified module genes highly associated with LFS using weighted gene co-expression network analysis (WGCNA). When the soft-thresholding power was set to 4 (scale-free *R*² = 0.9), the scale-free network exhibited optimal performance (Supplementary Fig. S1A). As shown in Fig. 2A, the blue module genes displayed a strong positive correlation with LFS (*r* = 0.65, *p* = 6 × 10⁻⁴). Consequently, 1410 genes from the LFS-associated blue module were selected as module genes for subsequent analyzes (Supplementary Fig. S1B).

Principal component analysis (PCA) analysis revealed variability among the multiple OS datasets (Supplementary Fig. S1C, D). After batch correction (Supplementary Fig. S1E, F), we used the “limma” package to identify 563 OS-associated differentially expressed genes (DEGs) (Fig. 2B, and Supplementary Fig. S1G). The intersection of DEGs for OS (*n* = 563) and modular genes for LFS (*n* = 1410) resulted in 51 LFS-OS shared genes, as shown in the Venn diagram (Fig. 2C, and Supplementary Table S1). Their expression trends across both diseases are shown in Supplementary Fig. S2A, B.

### Functional enrichment analysis of LFS-OS shared genes

To investigate the potential mechanisms underlying the susceptibility of LFS to OS, we performed Gene Ontology (GO) and Kyoto Encyclopedia of Genes and Genomes (KEGG) functional enrichment analyzes on the identified shared genes (Fig. 2D). In the biological processes (BP) category, primarily enriched in biological processes related to cell division, such as nuclear division, nuclear chromosome segregation, chromosome segregation. In the cellular components (CC) category, genes were mainly enriched in the chromosomal region, centromeric region, spindle, and chromosome centromeric region. For molecular functions (MF), the genes were predominantly associated with single-stranded DNA binding. To help us understand the similarities and differences between genes in different GO functional categories, we conducted clustering and dimensionality reduction on the enriched GO terms. The results showed that the terms were grouped into five main clusters: sister chromatid division separation, spindle assembly checkpoint signaling, nuclear division segregation phase, metaphase/anaphase organization of the cell cycle, and chromosome spindle checkpoint signaling (Fig. 2E, and Supplementary Fig. S2C). These findings suggest that the functions of the LFS–OS shared genes are primarily associated with cell division, chromosome segregation, and mitotic processes. The dysregulation of these functions are all closely related to the mechanism of cancer, particularly the pathogenesis of OS.

KEGG pathway analysis revealed that these genes were significantly enriched with the cell cycle pathway and also played important roles in signaling pathways such as DNA replication, oocyte meiosis and human T-cell leukemia virus 1 infection (Fig. 2D). Through dimensionality reduction of the KEGG enrichment, we found that its pathway is mainly associated with gap cell junction complex, DNA anemia excision repair, insulin nucleocytoplasmic drug resistance, cell FoxO human cycle (Fig. 2F, and Supplementary Fig. S2D). These findings suggest that cell cycle regulation and gene repair mechanisms may play a critical role in LFS and OS occurrence.

### Identification of feature genes via machine learning

**Fig. 3:**
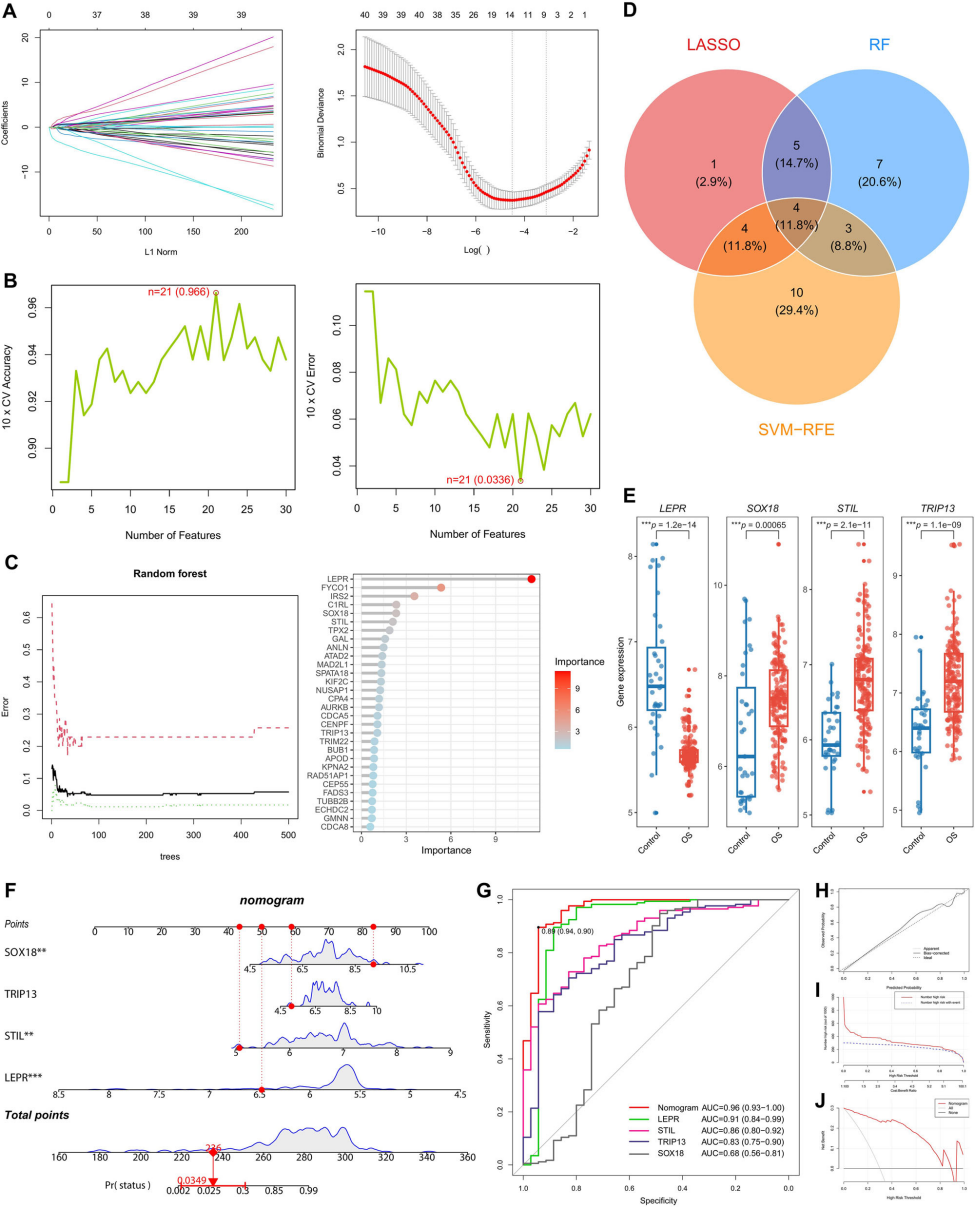
Machine-learning identification and validation of LFS–OS related feature genes. **A** Plot of LASSO coefficient profiles and partial likelihood deviance. **B** The SVM-RFE model identified 21 genes with the highest accuracy and lowest error. **C** Random forest modeling of genetic importance ranking based on MeanDecreaseGini. **D** Venn diagram integrating the three machine-learning algorithms. LEPR, SOX18, STIL, TRIP13 were consistently identified as robust LFS–OS feature genes. **E** Four feature genes exhibit significantly abnormal expression in OS samples (^***^p < 0.001). **F** Construction of a nomogram model integrating the four feature genes for predicting OS risk. **G** ROC curve analysis. The nomogram achieved the highest predictive performance (AUC = 0.96), outperforming individual genes (LEPR: 0.91; STIL: 0.86; TRIP13: 0.83; SOX18: 0.68). **H** Calibration curve demonstrating strong agreement between predicted and observed OS risk, indicating good model calibration. **I** Decision curve analysis showing the clinical net benefit of the nomogram across a range of threshold probabilities. **J** Clinical impact curve confirms the utility of nomogram model in stratified high-risk OS patients.

To identify feature genes associated with OS susceptibility, we used three machine learning algorithms: Random Forest (RF), Least Absolute Shrinkage and Selection Operator (LASSO), and Support Vector Machine–Recursive Feature Elimination (SVM-RFE) for feature selection. LASSO regression identified 14 candidate feature genes with the lowest binomial deviance (Fig. 3A). SVM-RFE results showed that the top 21 candidate feature genes exhibited the highest accuracy and lowest error (Fig. 3B). The RF algorithm identified 19 candidate feature genes with a MeanDecreaseGini > 1.0 (Fig. 3C). Finally, we finally identified four common feature genes, LEPR, SOX18, STIL, and TRIP13, by intersecting the candidate feature genes obtained from the three machine learning algorithms (Fig. 3D), all of which demonstrated strong predictive performance across different algorithms. The Wilcoxon test revealed that LEPR was downregulated in OS, whereas SOX18, STIL, and TRIP13 were upregulated (Fig. 3E, and Supplementary Fig. S3A). STIL and LEPR are located on chromosome 1, while TRIP13 and SOX18 are located on chromosomes 5 and 20, respectively (Supplementary Fig. S3B). A positive correlation was observed between TRIP13 and STIL, whereas LEPR showed negative correlations with both STIL and TRIP13, suggesting potential regulatory roles among these genes in OS pathogenesis (Supplementary Fig. S3C). Moreover, we found that STIL was connected to multiple key genes and played a critical role in centrosome assembly, centrosome duplication, and centrosome cycle regulation. Dysfunction of STIL may lead to aberrant centrosome replication, resulting in chromosomal instability and increased risk of cancer cell proliferation, indicating that it may serve as a core gene within this network (Supplementary Fig. S3D).

### Construction and validation of the nomogram

We performed logistic regression analysis based on the expression levels of four feature genes (SOX18, TRIP13, STIL, and LEPR) and constructed a personalized predictive nomogram model (Fig. 3F). According to 5-fold cross-validation, the AUC for the nomogram reached 0.96 (95% CI: 0.93–1.00), outperforming the individual predictive power of each feature gene, including LEPR (AUC = 0.91, 95% CI: 0.84–0.99), STIL (AUC = 0.86, 95% CI: 0.80–0.92), TRIP13 (AUC = 0.83, 95% CI: 0.75–0.90), and SOX18 (AUC = 0.68, 95% CI: 0.56–0.81) (Fig. 3G). The calibration curve demonstrated good agreement between predicted and observed outcomes, indicating favorable predictive performance (Fig. 3H). Additionally, decision curve analysis (DCA) and clinical impact analysis confirmed the strong clinical utility of the nomogram at a range of risk thresholds (Fig. 3I, J).

### SHAP interpretability

To assess the intrinsic classification potential of the identified 4 feature genes and to understand the contribution of each gene, we benchmarked its performance using ten machine learning algorithms and conducted 20-fold cross-validation. Among the models, GBM achieved the best classification performance with an AUC of 0.958 (Supplementary Fig. S4A). Other models, such as RF and SVM, also showed high AUC values (AUC = 0.950), further confirming the stability and reliability of these feature genes in OS prediction. To better understand the contribution of each feature gene to the predictive model, we applied the SHAP method to interpret and visualize the GBM model. Supplementary Fig. S4B shows that the LEPR gene has the highest SHAP value of 0.178, indicating its greatest importance in OS prediction. Data points to the right of the X-axis represent positive correlations with OS occurrence (risk factors), while those to the left of the X-axis represent negative correlations with OS occurrence (protective factors). The relationships and interactions between gene expression levels and SHAP values are illustrated in Supplementary Fig. S4C–F. Waterfall plots in Supplementary Fig. S4G, H.

### High STIL expression correlates with OS metastasis, poor prognosis, and antitumor immune suppression

**Fig. 4:**
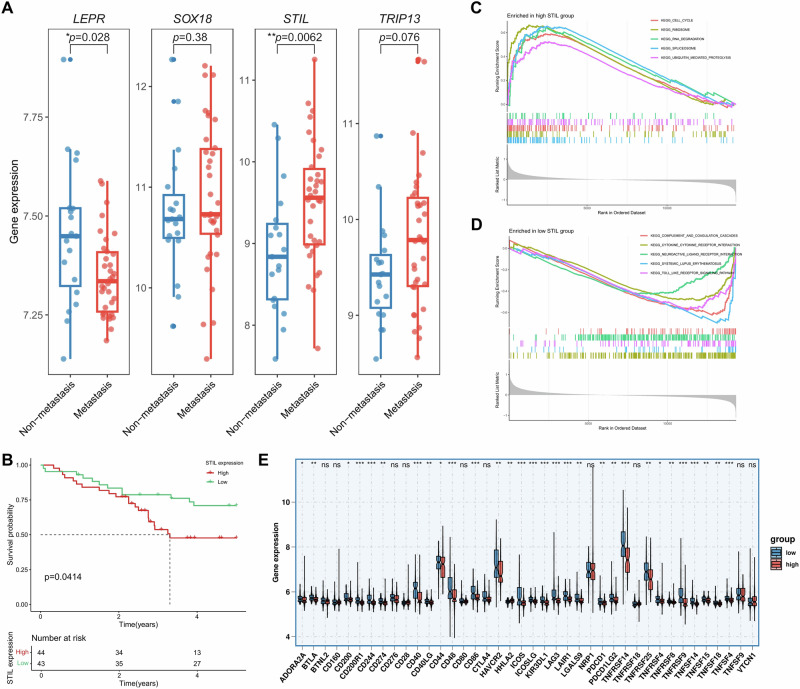
Clinical association, pathway enrichment, and immunophenotyping related to STIL expression in osteosarcoma. **A** Expression of LEPR, SOX18, STIL, and TRIP13 in metastatic and non-metastatic osteosarcoma. STIL and LEPR showed statistically significant upregulation in metastatic samples. **B** Kaplan–Meier survival analysis based on STIL expression levels. High STIL expression was significantly associated with poorer overall survival. **C**, **D** GSEA enrichment plots for STIL high- and low-expression. STIL high expression indicates significant enrichment of cell cycle-related pathways. STIL low expression preferentially enriches immune-related and differentiation-related pathways. **E** Differences in immune checkpoint genes expression between STIL high- and low-expression groups. Multiple co-stimulatory and co-inhibitory immune checkpoint molecules were significantly downregulated in the STIL high-expression group. This pattern indicates the presence of an “immune desert” characteristic.

To reveal whether the feature genes have clinical significantly, we compared their expression levels between metastatic and non-metastatic OS samples. STIL was significantly upregulated in metastatic OS samples (*p* < 0.01), while LEPR was downregulated in metastatic samples (*p* < 0.05) (Fig. 4A). Kaplan–Meier (KM) survival analysis showed that high STIL expression was significantly associated with poor prognosis (*p* < 0.05), whereas the expression levels of LEPR, SOX18, and TRIP13 were not significantly correlated with overall survival (Fig. 4B, and Supplementary Fig. S5A–C). Therefore, based on its unique and significant association with both metastasis and poor prognosis, STIL was selected as the key gene for all subsequent functional and mechanistic analyzes.

Gene Set Enrichment Analysis (GSEA) analysis showed that the STIL high-expression group was significantly enriched in pathways including cell cycle, ribosome, RNA degradation, spliceosome, and ubiquitin-mediated proteolysis, suggesting that high STIL expression may play an important role in tumor cell proliferation and growth (Fig. 4C). In contrast, the STIL low-expression group showed significant enrichment in pathways such as complement and coagulation cascades, cytokine–cytokine receptor interaction, neuroactive ligand–receptor interaction, and Toll-like receptor signaling pathway, indicating that low STIL expression may be associated with immune responses, inflammatory processes, and autoimmune pathologies (Fig. 4D). GSEA enrichment results for differential expression of LEPR, SOX18, and TRIP13 are shown in Supplementary Fig. S5D–I.

Immune infiltration analysis was performed on the merged OS dataset using the CIBERSORT algorithm, which obtained the relative proportions of 22 immune cell types in each sample and revealed differential immune cell expression between normal and OS tissues (Supplementary Fig. S6A, B). Compared with the control group, plasma cells, CD8⁺ T cells, gamma delta T cells, monocytes, resting mast cells, and neutrophils were significantly decreased in OS, while only naïve CD4⁺ T cells and M0 macrophages were upregulated in OS samples, suggesting the presence of immune suppression in the OS microenvironment. A correlation heatmap further illustrated the relationships among different immune cell types (Supplementary Fig. S6C).

In addition, integration of feature genes expression with immune cell infiltration data revealed significant correlations between feature genes and specific immune cell types (Supplementary Fig. S6D). STIL exhibited strong positive correlations with resting CD4 memory T cells, M0 macrophages, plasma cells, and resting NK cells, while showing negative correlations with follicular helper T cells, CD8⁺ T cells, gamma delta T cells, and regulatory T cells (Tregs). These findings suggest that STIL may influence the progression of OS by modulating the immune microenvironment (Supplementary Fig. S6E). Correlations between LEPR, SOX18, and TRIP13 and immune cells are shown in Supplementary Fig. S6F–H.

Analysis of ICGs revealed that expression of both key immune checkpoint inhibitor targets (e.g., PD-1, PD-L1, CTLA4) and co-stimulatory pathways (e.g., CD40, CD86, ICOS) was significantly downregulated in the STIL-high expression group (*p* < 0.05; Fig. 4E). This indicates that STIL-high tumors foster an immunologically “cold” or “immune-desert” microenvironment.

### Construction of a cell atlas for OS

**Fig. 5:**
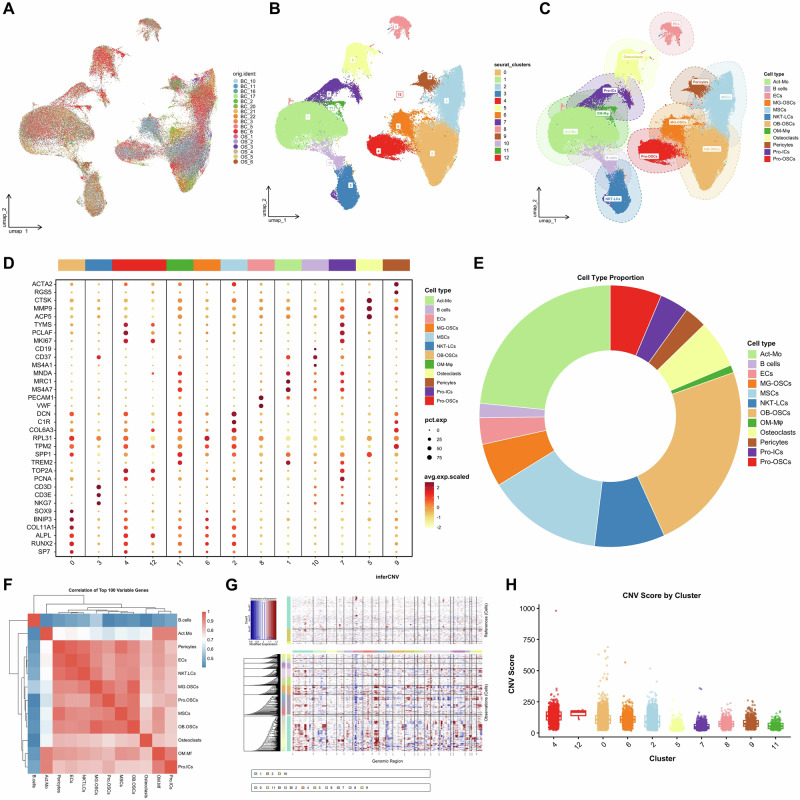
Single-cell transcriptomic landscape of osteosarcoma and identification of major malignant cell populations. **A** UMAP plot of the multicellular ecosystem of 17 tissue samples. **B** UMAP plot showing 13 distinct cell clusters identified through cluster analysis. **C** UMAP plot of 12 cell clusters in the OS. **D** The marker genes of each cell cluster. Dot size represents the percentage of cells expressing the marker, while color intensity reflects average scaled expression. **E** Annotate the proportion of cell types in the OS single-cell dataset. **F** Heatmap of correlations between cell types for the top 1000 highly variable genes, malignant OS clusters (Pro-OSCs, OB-OSCs, MG-OSCs) show high transcriptional similarity. **G** Heatmap of copy number variation (CNV) profiles in different cell clusters. Malignant OS cell clusters exhibit extensive chromosomal gains and losses. **H** Boxplot of CNV scores for different cell clusters. Cluster 4, 12,0,6 (Pro-OSCs, MG-OSCs and OB-OSCs) show the highest CNV scores, confirming their malignant status.

We integrated two scRNA-seq datasets, GSE162454 and GSE152048, obtaining a total of 17 OS samples. After data preprocessing and batch effect correction (Supplementary Fig. S7A, B), we constructed a high-quality single-cell atlas of OS comprising 118,206 cells (Fig. 5A). Clustering analysis using parameters dims = 1:10 and resolution = 0.2 identified 13 distinct cell clusters (Fig. 5B, and Supplementary Fig. S8A, B). Based on representative marker genes, we annotated the cell clusters into 12 major cell types (Fig. 5C), including: Osteomimicry Macrophages (OM-Mφ): IBST, TREM2, SPP1, MNDA; Osteoblastic Osteosarcoma Cells (OB-OSCs): COL11A1, BNIP3, SOX9, ACAN, COL2A1; Myogenic Osteosarcoma Cells (MG-OSCs): TNNT2, MYH3, MYOG, MYL1; Proliferating Immune Cells (Pro-ICs): HLA-DRA, HLA-DPB1, PCNA, PCLAF, MKI67; Proliferative Osteosarcoma Cells (Pro-OSCs): PCNA, MKI67, TOP2A, RUNX2; Mesenchymal Stem Cells (MSCs): SFRP2, CXCL12, MME; Activated Monocytes (Act-Mo): CD14, MRC1, MNDA, CTSS, S100A9; Osteoclasts: ACP5, MMP9, CTSK; B cells: MS4A1, CD79A, CD19; NKT-like cells: NKG7, TRAC, CD3E, CD3D; Endothelial cells: VWF, PECAM1, EGFL7, PLVAP; and Pericytes: RGS5, ACTA2. The full list of marker genes is shown in Fig. 5D. The cellular composition within OS tissues was quantified as shown in Fig. 5E.

Subsequently, we performed GSVA analysis for each cell cluster using the Hallmark gene sets and generated a heatmap of the top 50 enriched pathways (Supplementary Fig. S9A). Notably, the “Feature genes” custom-defined in our study also ranked among the top 5 (Supplementary Fig. S9B).

A cellular correlation heatmap was further drawn based on the average expression levels of highly variable genes, and the results showed that there were significant expression differences among the cells (Fig. 5F). MG-OSCs, Pro-OSCs and OB-OSCs clustered into one group, showing strong expression consistency, suggesting that the three may constitute a continuous spectrum in the process of OS development. In contrast, B cells displayed a distinct expression profile compared to other cell types, indicating a functionally independent identity. A degree of correlation was also observed between OM-Mφ and Pro-ICs, implying potential co-involvement in immune regulation.

CNV analysis was performed on unannotated cells using inferCNV, and malignancy scores were calculated for each cell cluster (Fig. 5G, H). The results showed that cells in Clusters 12, 0, 4, and 6 exhibited significant copy number variations, indicating their potential malignancy. This finding was highly consistent with the clustering of the three OSC subtypes (OB-OSCs, MG-OSCs, and Pro-OSCs) in Fig. 5F. Subsequent analyzes focused on these three malignant cell populations.

### STIL is highly expressed in the highly malignant cell subclusters in the early stage

**Fig. 6:**
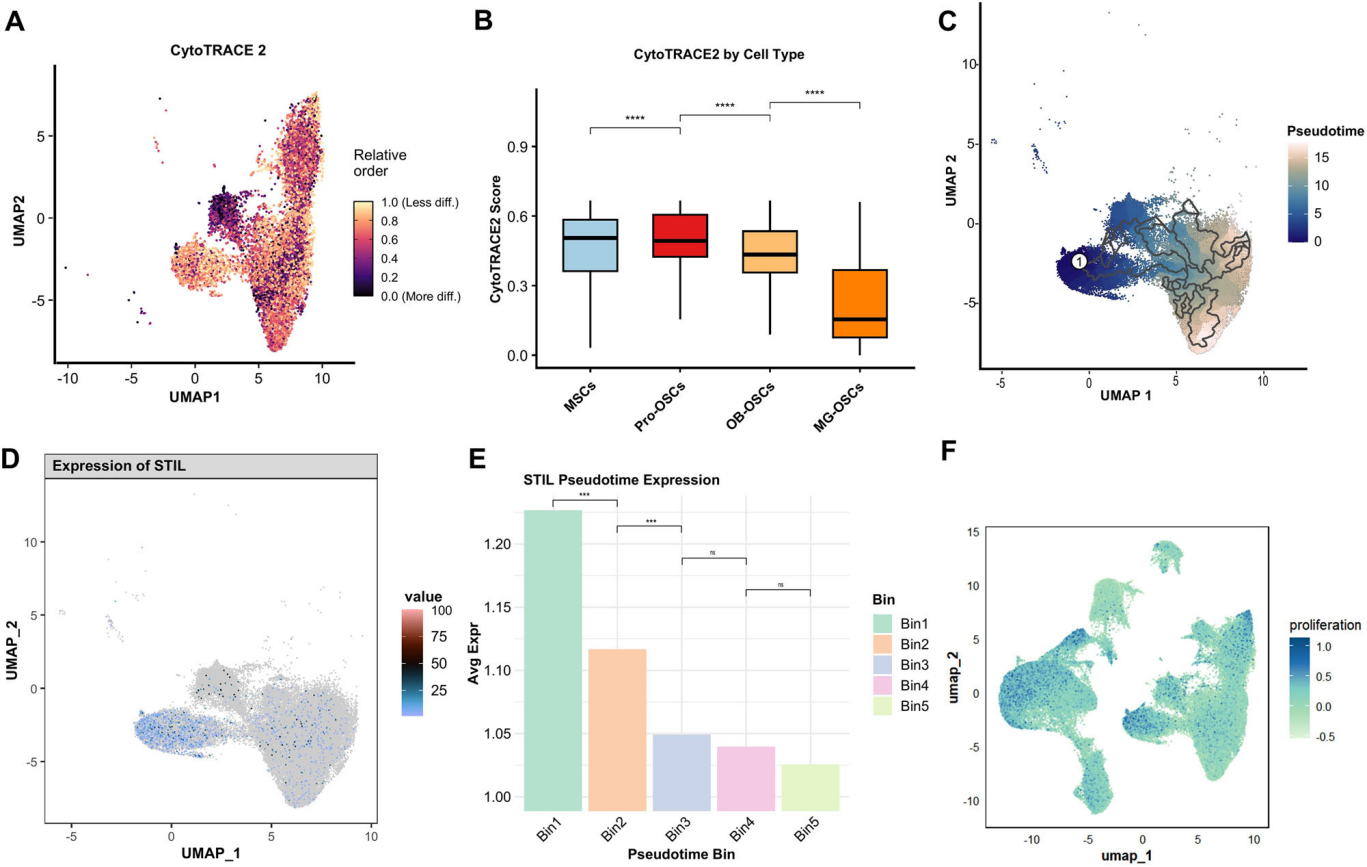
Stemness scoring, developmental trajectory, and STIL expression dynamics in malignant osteosarcoma cells. **A** UMAP plot of stemness scores for cell clusters based on the CytoTRACE2 algorithm. **B** CytoTRACE2 scores in major malignant subclusters. Pro-OSCs exhibit the highest stemness scores. **C** Pseudotime trajectory analysis of malignant cell subclusters. Pro-OSCs are located at the beginning of trajectory. **D** UMAP plot displays the expression levels of STIL in malignant cell subclusters. **E** Average STIL expression across pseudotime bins (Bin1–Bin5). STIL expression is significantly elevated in early pseudotime and progressively decreases along differentiation. **F** Proliferation signature mapped onto UMAP space. High-proliferation regions overlap with high STIL expression and CytoTRACE2-defined stem-like populations, reinforcing the link between STIL, proliferative activity, and early OS cell states. (^***^p < 0.001, ^****^p < 0.0001, ns = not significant).

To investigate the potential association between STIL and the stemness and developmental state of malignant cells, we employed the CytoTRACE algorithm to perform stemness scoring on OB-OSCs, MG-OSCs, and Pro-OSCs. Given their inherent high stemness, MSCs were included as a control group. Results revealed that Pro-OSCs exhibited significantly higher stemness scores and differentiation potential compared to other malignant cell subclusters (Fig. 6A, B).

Further pseudotime analysis of Pro-OSCs, OB-OSCs, and MG-OSCs was performed to assess their developmental dynamics. Results showed that Pro-OSCs were located at the beginning of the trajectory (Fig. 6C). The UMAP plot of trajectory embedding revealed that STIL was predominantly expressed in Pro-OSCs (Fig. 6D). Pseudotime analysis of STIL expression demonstrated its elevated expression during early developmental stages (Fig. 6E). Proliferation scoring of OS cells further indicated high proliferative activity in Pro-OSCs, suggesting STIL may participate in their proliferation regulation and play a critical role in OS progression (Fig. 6F). The distribution and pseudo-time analysis of the remaining feature genes (SOX18, TRIP13, and LEPR) within the malignant cell clusters are shown in Supplementary Fig. S10.

### STIL high expression influences cell communication patterns of Pro-OSCs and tumor microenvironment

**Fig. 7:**
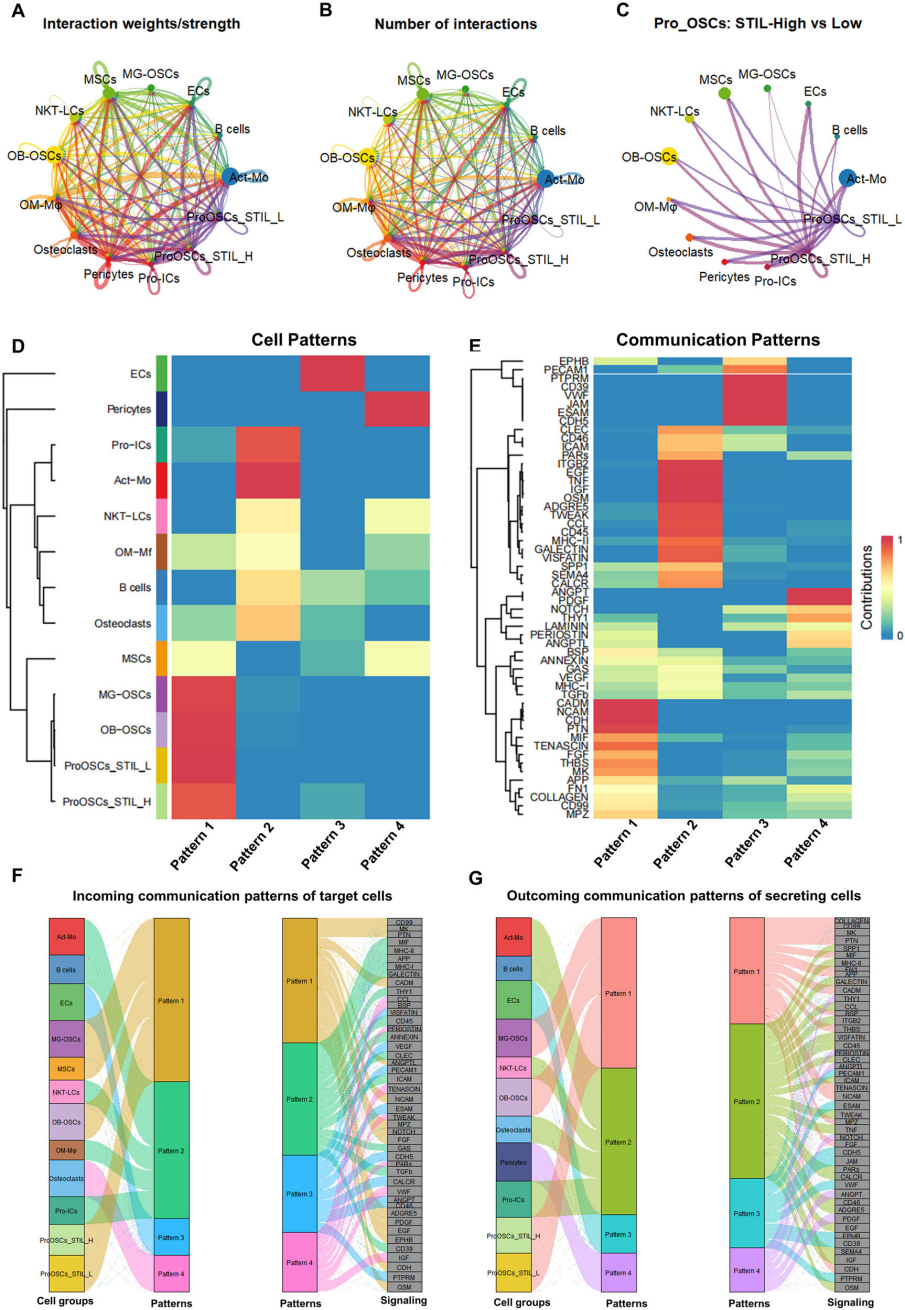
STIL expression reprograms communication patterns of Pro-OSCs and osteosarcoma microenvironment. **A** Network of the strength of cell communication interactions. **B** Network of number of cell communication interactions. **C** Network of the strength and number of cell communication interactions between Pro-OSCs-STIL-H and Pro-OSCs-STIL-L subclusters. **D** Heatmap of clustering of cell patterns for cell communication. OB-OSCs, MG-OSCs, Pro-OSCs-STIL-High and Pro-OSCs-STIL-Low cluster together in Pattern 1, suggesting shared signal-receiving characteristics among osteogenic malignant populations. **E** Signaling contribution heatmap showing that Pattern 1 is enriched for multiple key ligand–receptor programs. **F** The river plot of incoming communication patterns of target cells. **G** The river plot of outgoing communication patterns of secreting cells.

We divided Pro-OSCs into two subclusters into STIL-high (Pro-OSCs-STIL-H) and STIL-low (Pro-OSCs-STIL-L) based on the median expression level of STIL within the Pro-OSCs cluster. On this basis, we investigated how STIL expression affects cell communication patterns between Pro-OSCs and other clusters. The results revealed significant differences in both communication strength and the number of interactions between the STIL-high and STIL-low subclusters and other clusters (Fig. 7A–C). Considering the critical importance of communication directionality in functional regulation, we further generated circle diagrams illustrating outgoing interactions from sender clusters to the Pro-OSCs-STIL-High and STIL-Low subclusters (Supplementary Fig. S11). These results suggest that high STIL expression not only reshapes the signal reception profile of Pro-OSCs but also significantly alters incoming signals from immune, osteogenic, and stromal clusters. We further applied non-negative matrix factorization (NMF) to cluster the cell communication patterns. OB-OSCs, MG-OSCs, STIL-high, and STIL-low were all classified into Pattern 1, suggesting that they are highly similar (Fig. 7D). Contribution analysis indicated that Pattern 1 was enriched in several key signaling pathways, including CADM, NCAM, MIF, PTN, APP, and FN1 (Fig. 7E), implying that osteogenic-related clusters may cooperatively respond to these signals under specific pathological conditions, thereby participating in tumor development and bone microenvironment regulation.

Subsequently, we performed clustering analysis of both incoming and outgoing communication patterns. The river plot showed that Pattern 1 was predominantly active in the incoming communication pattern of signals related to CD99, FN1, and PTN pathways (Fig. 7F), while outgoing communication pattern signals were mainly associated with MIF, APP, and MPZ pathways (Fig. 7G). These results suggest that Pro-OSCs with high STIL expression not only play a key role in incoming communication pattern but may also actively participate in sending signals, thereby modulating the communication network of the tumor microenvironment and influencing the biological progression of OS.

### Analysis of heterogeneity in cell communication networks among STIL-based Pro-OSCs subclusters

To investigate the impact of STIL expression on the cell communication function of Pro-OSCs, we selected four key signaling pathways, MIF, FN1, APP, and PTN, to analyze the heterogeneity between the STIL-high and STIL-low subclusters within the cell communication network.

**Fig. 8:**
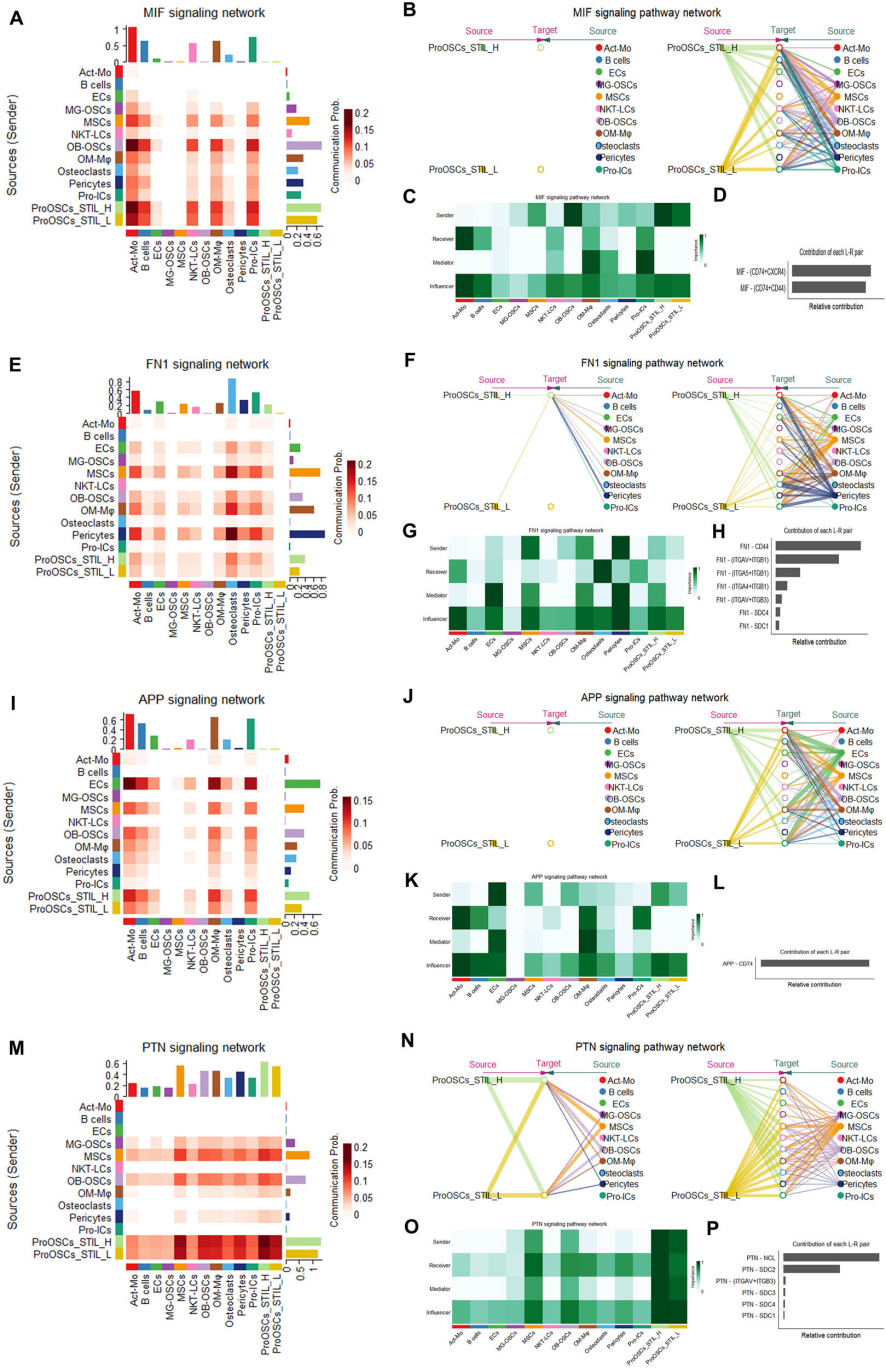
STIL-high and low regulate key signaling pathways (MIF, FN1, APP, PTN) in Pro-OSCs. Heatmaps of MIF (**A**) FN1 (**E**) APP (**I**) and PTN (**M**) signaling pathways in cell clusters. Rows and columns represent sender and receiver clusters, respectively. Hierarchical plot of cell clusters for MIF (**B**) FN1 (**F**) APP (**J**) and PTN (**N**) signaling pathways. Circle size corresponds to the proportion of cells in each cluster. Edge width reflects interaction strength. In the left panel, the central target is the selected receiver cluster; in the right panel, target refers to all other clusters aside from the selected one. Heatmap of the importance of cell clusters as a sender, receiver, mediator and influencer in the MIF (**C**) FN1 (**G**) APP (**K**) and PTN (**O**) signaling pathways. Bar graph of relative contribution of ligand-receptor (**L**–**R**) hierarchy in MIF (**D**) FN1 (**H**) APP (**L**) and PTN (**P**) signaling pathways.

In the MIF signaling pathway, the STIL-high subcluster showed significantly enhanced communication with immune clusters such as Act-Mo, B cells, NKT-LCs, and Pro-ICs (Fig. 8A), primarily functioning as a signal sender (Fig. 8B). Centrality analysis revealed that the STIL-high subcluster scored higher in both sender and influencer roles (Fig. 8C), with MIF–(CD74+CXCR4) identified as the highest contributing ligand–receptor pair (Fig. 8D). In the FN1 signaling pathway, communication between the STIL-high subcluster and osteoclasts was markedly increased (Fig. 8E), with dual roles as both sender and receiver observed in the network (Fig. 8F). And the centrality scores of STIL-high were significantly elevated in both mediator and influencer roles (Fig. 8G), and FN1–CD44 was identified as the dominant communication axis (Fig. 8H). In the APP signaling pathway, communication activity between STIL-high and multiple immune clusters was enhanced (Fig. 8I), and it primarily acted as a signal sender (Fig. 8J). And centrality analysis showed increased sender and influencer scores (Fig. 8K), with APP–CD74 identified as the key ligand–receptor pair (Fig. 8L). In the PTN signaling pathway, enhanced communication was observed between STIL-high and MSCs as well as other malignant clusters (Fig. 8M), with STIL-high functioning as both sender and receiver (Fig. 8N), suggesting autocrine potential. And the centrality scores for multiple functional roles were significantly increased (Fig. 8O), with PTN–NCL emerging as the highest contributing ligand–receptor pair (Fig. 8P).

To further investigate the impact of STIL on the tumor immune microenvironment, we analyzed ligand–receptor interactions between Pro-OSCs-STIL-H or STIL-L subclusters and immune-related clusters (Supplementary Fig. S12). These provide that STIL expression may actively reshape intercellular signaling with immune components, thereby promoting immune escape.

### STIL exhibits differential effects on OS in different TP53 states

**Fig. 9:**
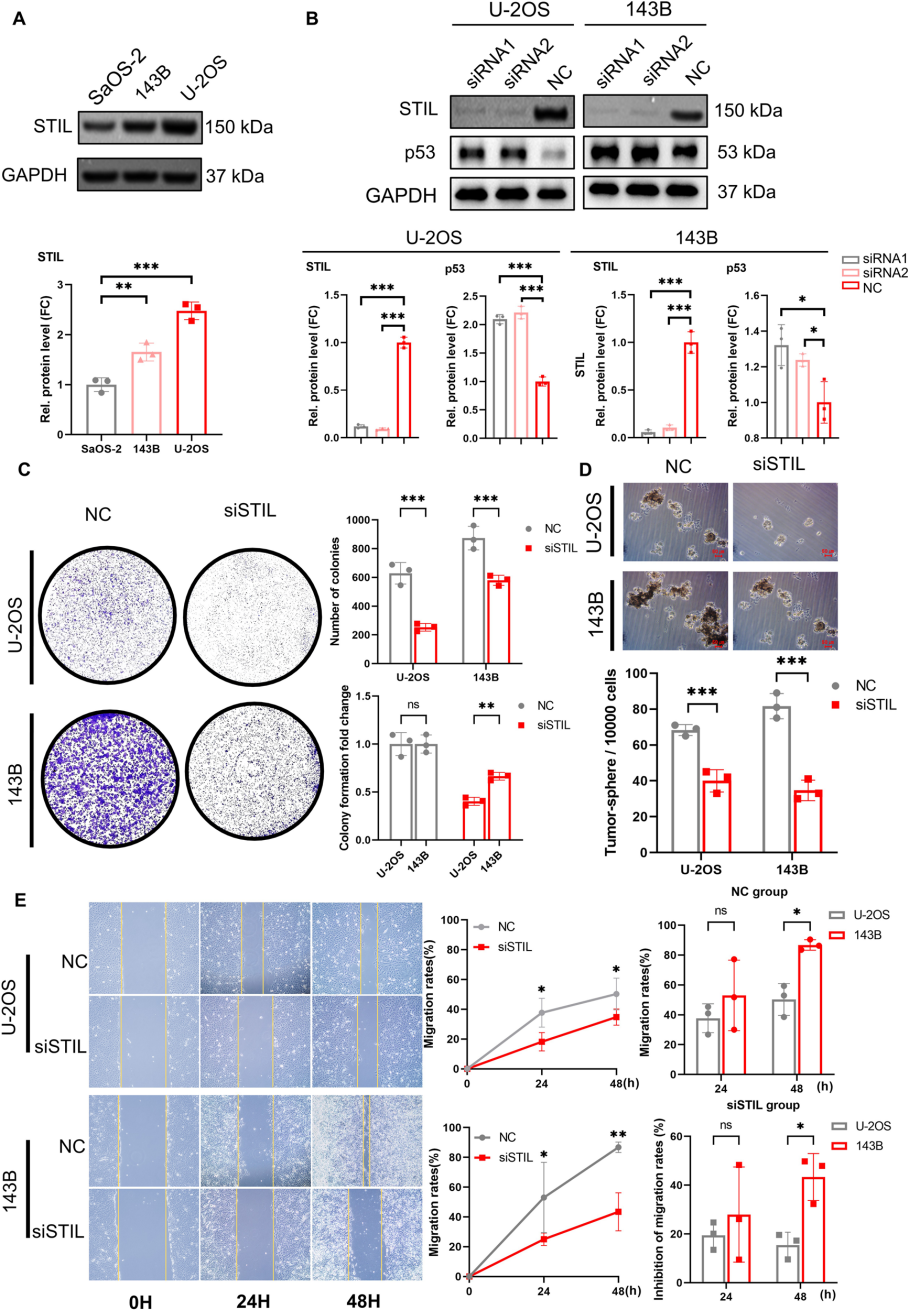
Experimental validation reveals STIL negatively regulates p53 stability and differentially drives malignant phenotypes in osteosarcoma cells. **A** Western blot analysis of STIL protein expression levels in SaOS-2, 143B (TP53-mutant), and U-2 OS (TP53-wildtype) cell lines. **B** Western blot analysis validating the knockdown efficiency of small interfering RNA (siRNA) targeting STIL and its effect on p53 protein levels in U-2 OS and 143B cells. Knockdown of STIL resulted in a significant upregulation of p53 protein in both wild-type and mutant cell lines. **C** Colony formation assay assessing the effect of STIL knockdown on cell proliferation. The upper panel displays representative images and colony counts; the lower panel shows the fold change in colony formation relative to the negative control (NC). U-2 OS cells exhibited a more pronounced inhibition of proliferation (lower fold change) compared to 143B cells. **D** Tumor sphere formation assay evaluating the role of STIL in maintaining stemness. Representative images (top) and quantification (bottom) demonstrate that STIL knockdown significantly impairs the sphere-forming ability of both U-2 OS and 143B cells. **E** Wound healing assay assessing cell migration capabilities at 0, 24, and 48 h. While 143B cells showed a higher basal migration rate in the NC group, the inhibition rate analysis (bottom right) reveals that 143B cells are significantly more sensitive to migration inhibition by STIL knockdown at 48 h compared to U-2 OS cells. Data are presented as mean ± SD (^*^p < 0.05, ^**^p < 0.01, ^***^p < 0.001, ns = not significant).^***^p < 0.001, ns = not significant).

To validate the biological function of STIL in LFS-OS and investigate its potential association with TP53 status, we conducted in vitro validation using cell lines. First, we assessed the basal expression levels of STIL in three OS cell lines (SaOS-2, 143B, U-2 OS). Western blot analysis revealed that STIL was highly expressed in both 143B (TP53 mutant, mimicking the LFS phenotype) and U-2 OS (TP53 wild-type) cells compared to SaOS-2 (TP53-deficient) (Fig. 9A). Consequently, we selected the 143B and U-2 OS cell lines for subsequent functional experiments.

Following transfection of 143B and U-2 OS cells with si-STIL, western blot analysis confirmed a significant reduction in STIL protein levels in both cell lines. Interestingly, we observed that following STIL knockdown, p53 protein expression was upregulated in both wild-type and mutant cells (Fig. 9B), suggesting that STIL may negatively regulate p53 at the protein level.

Subsequently, we evaluated the impact of STIL on cell proliferation using a colony formation assay. Results demonstrated that STIL knockdown significantly reduced the number of colonies formed in both cell lines. Notably, despite the faster basal growth rate of 143B cells, calculation of fold change revealed that TP53 wild-type U-2 OS cells exhibited greater sensitivity to STIL knockdown, with a more pronounced proliferation inhibition effect (Fig. 9C). This suggests that in the context of functional TP53, STIL deficiency may more readily trigger cell cycle arrest.

To validate the relationship between STIL and tumor stemness identified in single-cell analysis, we performed a tumor sphere formation assay. Consistent with bioinformatics predictions, STIL knockdown significantly inhibited the sphere-forming capacity of U-2 OS and 143B cells (Fig. 9D), confirming STIL’s critical role in maintaining OS cell stemness characteristics.

Finally, considering that LFS patients often carry a high risk of metastasis, we evaluated the impact of STIL on cell migration capacity using a wound healing assay. Results showed that STIL knockdown delayed the healing speed in both cell lines. However, although 143B cells exhibited faster migration rates than U-2 OS cells in the control group (NC), the migration inhibition rate of 143B cells in the STIL knockdown group was significantly higher than that of U-2 OS cells at 48 h (Fig. 9E). This finding holds significant clinical implications, suggesting that STIL is crucial for maintaining the highly invasive and metastatic properties of TP53-mutant (LFS-associated) OS cells.

### Drug sensitivity and pan-cancer analysis of STIL

We analyzed the association between STIL expression and drug sensitivity. By calculating the half-maximal inhibitory concentration (IC50) values of various anti-cancer drugs between STIL-high and STIL-low expression groups, we found that the expression level of STIL was significantly correlated with the responsiveness to multiple drugs (Supplementary Fig. S13A). Samples with high STIL expression showed greater sensitivity to several agents, including Afatinib, WEE1 Inhibitor, Paclitaxel, and Carmustine, while low STIL expression was associated with increased sensitivity to drugs such as Selumetinib, Trametinib, and Ribociclib. These findings suggest that STIL expression may differentially influence the response to different types of anti-cancer drugs, which provides a basis for the potential application of targeted STIL for personalized cancer therapy.

Pan-cancer analysis revealed that STIL expression was significantly higher in tumor tissues than in normal tissues across most cancer types (28/33) (Supplementary Fig. S13B). This indicates that STIL may function as a cancer-associated gene involved in the initiation and progression of most tumors. Furthermore, Cox regression analysis revealed that high STIL expression was associated with poor prognosis in most cancers, including overall survival, disease-specific survival, disease-free interval, and progression-free interval (Supplementary Fig. S13C). It is suggested that STIL is not only a potential biomarker of tumorigenesis but also may serve as an important predictor of prognosis in cancer patients. Overall, these results suggest that high expression of STIL in a variety of cancer types is closely associated with poorer clinical prognosis and that STIL has a good potential for clinical application as a prognostic marker for cancer.

## Discussion

OS is the most common primary malignant bone tumor in adolescents, characterized by high invasiveness and strong metastatic potential, which leads to the survival of patients with OS remains a great challenge. Since OS accounts for less than 1% of malignancies, scientists have hypothesized that it may be associated with genetic susceptibility syndromes^19^. Among them, LFS is a genetic susceptibility syndrome associated with germline TP53 variants and is one of the most frequently detected syndromes in patients with OS^9,20^. TP53 mutations lead to dysfunctional DNA repair, reduced tumor suppression function, etc., and previous studies have confirmed that germline TP53 mutations significantly increase susceptibility to OS and lead to poorer survival^21^. However, whether LFS contributes to OS through additional molecular pathways remains unclear. This study integrates multi-omics data, machine learning, single-cell sequencing, and in vitro experimental validation to systematically reveal the pivotal role of STIL in LFS to OS.

We found that STIL belongs to a gene module highly associated with LFS and is significantly upregulated in OS, particularly in metastatic OS samples. Its high expression was markedly correlated with shorter patient survival and was mainly involved in key oncogenic pathways such as cell cycle regulation, mitosis, and centriole assembly. Moreover, STIL demonstrated strong discriminatory power in classification models, and SHAP analysis indicated a positive relationship between its expression level and the predicted probability of disease, further supporting its potential as a key feature gene. Importantly, we found that STIL knockdown significantly upregulates p53 protein levels. Furthermore, in TP53-mutant cells, STIL is essential for stemness maintenance and metastatic potential, highlighting its role in exploiting the mutant background to drive LFS-associated OS.

STIL encodes a cytoplasmic protein in the human genome that is primarily involved in centrioles biosynthesis. It plays a critical role in the regulation of the mitotic spindle checkpoint and is essential for proper cell cycle control^22^. Current studies have shown that STIL overexpression exerts oncogenic effects and is associated with various cancers and poor prognosis, including non-small cell lung cancer, bladder cancer, and T-cell acute lymphoblastic leukemia^23–26^. STIL is upregulated in late G1 phase and highly expressed during the S phase, promoting the G1/S transition and thereby enhancing cell division^27^. Moreover, STIL overexpression can activate excessive PLK4, leading to centrosome overduplication^23^. These alterations collectively contribute to the formation of multipolar spindles, chromosomal missegregation, and genomic instability, which may ultimately result in DNA damage and tumorigenesis.

To investigate the role of STIL in the specific genetic context of LFS, we compared TP53 wild-type (U-2 OS) and TP53 mutant (143B, mimicking LFS) cell lines in in vitro experiments. Our western blot results revealed a key molecular mechanism: following STIL knockdown, p53 protein levels significantly increased in both wild-type and mutant cells. This suggests STIL may function as a negative regulator of p53. TP53 is one of the most critical tumor suppressor genes in the human genome and is often referred to as the “guardian of the genome” due to its essential role in maintaining genomic stability^28^. The p53 protein encoded by TP53 can activate CDKN1A to promote the expression of p21 protein, thereby halting the cell cycle at the G1 phase and preventing entry into the S phase^29^. This arrest is crucial for DNA repair, as it allows time to repair DNA damage before replication. Moreover, co-occurrence of high STIL expression and TP53 mutations has been observed in some cancers. For example, in hepatocellular carcinoma, STIL overexpression is closely associated with poor prognosis, and frequent TP53 mutations are also present^30,31^. In our colony formation assay, TP53 wild-type U-2 OS cells exhibited a more pronounced proliferation inhibition following STIL knockdown compared to 143B cells. This may be because, in the wild-type context, STIL deficiency lifts the inhibition on p53, restoring the p53-mediated cell cycle “braking” mechanism and thereby causing a sharp halt in proliferation.

However, in TP53-mutated 143B (LFS model) cells, where p53 has lost its tumor-suppressing function, the oncogenic effects of STIL may no longer primarily depend on inhibiting p53 activity. Instead, they may shift toward maintaining tumor stemness and invasiveness. By scRNA-seq analysis, we identified multiple malignant cell clusters within OS samples, among which STIL was highly expressed mainly in the Pro-OSCs cluster. CytoTRACE scoring and pseudotime analysis revealed that Pro-OSCs predominantly reside in early developmental stages and exhibit high pluripotency, suggesting their potential identity as tumor-initiating cells. Our in vitro sphere formation assay confirmed that STIL knockdown significantly suppressed the spheroid-forming capacity of 143B and U-2 OS cells, demonstrating that STIL is an essential factor for maintaining OS cell stemness characteristics.

More importantly, STIL is closely associated with the high metastatic risk in OS patients. In our wound healing assay experiments, although TP53-mutant 143B cells exhibited stronger basal migration capacity than U-2 OS cells, they were more sensitive to STIL knockdown, demonstrating a higher migration inhibition rate. This indicates that the high metastatic potential driven by TP53 mutations is highly dependent on the sustained expression of STIL. This mechanism may relate to pathways identified in our single-cell communication analysis: in Pro-OSCs, high STIL expression was found to activate the PTN–NCL signaling pathway. PTN, a well-known stemness-regulating factor, has been shown in various tumors to maintain an undifferentiated state through autocrine mechanisms^32,33^, suggesting the formation of a stemness self-sustaining loop that facilitates the self-renewal and expansion of early tumor cells. Additionally, STIL activated the FN1–CD44 signaling pathway, and intercellular communication between STIL-high tumor cells and osteoclasts was strengthened, indicating a possible role in promoting bone resorption^34^. This osteolytic activity not only creates structural space for tumor invasion and dissemination but also releases bone-derived factors such as TGF-β and IGF, which are known to support stemness maintenance, thereby further reinforcing the stem-like state. Therefore, STIL’s oncogenic function in the OS not only involves intracellular cycle regulation and stemness maintenance, but also reshapes the tumor microenvironment by modulating external signaling pathways, thereby creating favorable conditions for tumor self-renewal, immune evasion, and distant metastasis^35^. Moreover, the elevated p53 protein levels observed in 143B may reflect severe mitotic stress induced by STIL deficiency. We hypothesize that in the LFS context, STIL undergoes a functional shift: transforming from a simple cell cycle regulator into a key factor that maintains tumor stemness and drives metastasis by activating signaling bypasses such as PTN-NCL and FN1-CD44. This mechanism explains why, in LFS patients with p53 dysfunction, high STIL expression remains strongly associated with poor prognosis and high metastatic risk.

In addition to regulating tumor cell plasticity and proliferation, STIL also plays a role in reshaping the tumor immune microenvironment. Our immune infiltration analysis revealed that compared to normal tissue, OS samples exhibited a significant reduction in anti-tumor immune cells such as CD8+ T cells and γδ T cells, while immune-suppressive cells like M0 macrophages were markedly increased. High expression of STIL suppresses the expression of ICI-related targets such as PD-1 and PD-L1. The expression of molecules crucial for T cell activation, including CD40, CD86, and ICOS, is also significantly downregulated. This indicates that the tumor microenvironment lacks the essential signals required to effectively initiate an anti-tumor immune response. Furthermore, the immune molecular targets upon which drugs rely for their effects are also absent. This suggests that high STIL expression may weaken immune surveillance mechanisms by regulating immune cell distribution or functional states, creating an “immune desert” that helps tumor cells evade immune clearance. Cell communication analysis further confirmed that the Pro-OSC subpopulation with high STIL expression enhances interactions with immune cells via the MIF-(CD74+CXCR4) axis, potentially contributing to the formation of an immunosuppressive microenvironment and promoting tumor escape from immune surveillance^36,37^. MIF, as an immunoregulatory factor, has been shown to drive the co-evolution of inflammatory microenvironments and tumors in various cancer types^38^. STIL may provide a survival advantage to OS cells by activating this pathway. Meanwhile, the APP–CD74 signaling pathway was also activated, suggesting that STIL may regulate neuron-like pathways to enhance immune evasion and cell survival^39^. Although APP has been less studied in OS, evidence from gliomas and other nervous system tumors indicates its involvement in CD74-mediated immunoregulatory signaling^40^. These findings suggest that STIL may play a crucial role in establishing a supportive microenvironment.

Although STIL is central to our study, other characteristic genes identified by machine learning models, TRIP13, SOX18, and LEPR, also constitute a key molecular network associated with LFS-related OS. Similar to STIL, TRIP13 is an AAA+ ATPase involved in mitotic checkpoint regulation, their co-upregulation suggests synergistic disruption of mitotic fidelity in LFS-OS^41^. As a transcription factor, SOX18 is known to participate in angiogenesis and embryonic development, potentially playing a role in supporting tumor microenvironment vascularization^42^. LEPR is the only downregulated gene, possibly suggesting metabolic reprogramming or altered cytokine signaling^43^. However, only STIL demonstrated a dual significant correlation with both metastatic status and poor survival. This indicates that while other genes contribute to the composition of disease characteristics, STIL is a more critical determinant of prognosis in LFS-associated OS.

Furthermore, our research indicates that STIL is not an oncogene exclusive to OS. In fact, our pan-cancer analysis confirms that STIL is upregulated in as many as 28 cancer types and is associated with a poor prognosis, further supporting its potential as a significant therapeutic target and prognostic factor. However, this does not diminish its particular significance in the context of this study. LFS is defined by germline TP53 mutations, which compromise the cell’s primary defense against genomic instability. Drug sensitivity analysis indicated that STIL-high expression OS cells was more sensitive to WEE1 inhibitor, afatinib, and paclitaxel, suggesting its potential as a therapeutic target for these treatment regimens. In particular, WEE1 Inhibitors have demonstrated promising therapeutic potential in TP53 mutant and deficient^44,45^. In the LFS context, TP53 mutations disrupt G1/S checkpoint function. Cells lacking functional G1 checkpoints become highly dependent on the WEE1-regulated G2/M checkpoint for DNA repair prior to entering mitosis. Consequently, in cells with TP53 mutations and STIL overexpression, inhibiting WEE1 eliminates this final protective mechanism, triggering massive centrosome duplication. This allows cells carrying unrepaired DNA to enter mitosis, ultimately leading to cell death. This phenomenon is also known as “mitotic catastrophe”^46–48^. Although the association between STIL status and the predictive sensitivity of some drugs is intriguing, its clinical efficacy remains unproven.

To our knowledge, this study is the first to systematically reveal the potential role of STIL in the pathogenesis of LFS and OS. However, our research still has several limitations. First, due to the low incidence of OS and LFS, the number of available samples is limited, which may affect the generalizability and robustness of the machine learning models. Second, this study specifically focuses on the association between LFS and OS. We did not perform a comparative analysis with other LFS-associated cancers. STIL may also contribute to the development of other tumors within the LFS spectrum, representing an important direction for future research. Furthermore, although we observed in vitro that inhibiting STIL leads to a increase in mutant p53 protein levels, this study has not yet elucidated the specific molecular mechanisms by which STIL regulates p53 protein stability, nor has it fully clarified how this regulation specifically triggers downstream signaling cascades in the LFS context. Furthermore, since LFS is a systemic syndrome, the cell line models we employed cannot fully replicate the complex tumor microenvironment observed in human patients. In addition, the high heterogeneity of OS and the pseudotime assumptions inherent in single-cell trajectory analysis limit the ability to precisely define the sequential role of STIL during tumor progression. Therefore, future studies involving larger clinical cohorts and experimental validation are needed to further confirm the oncogenic mechanisms of STIL and evaluate its potential as a therapeutic target.

In summary, this study integrates multi-omics analysis and in vitro experiments to reveal the pivotal role of STIL in promoting OS growth and metastasis in the context of TP53 mutations. Highly expressed in malignant cells with strong stem-like properties, STIL drives tumor progression through multiple mechanisms: suppressing p53 levels, maintaining tumor stemness via multiple pathways, promoting tumor growth, and regulating immune evasion. These findings provide new insights into the molecular mechanisms underlying OS development in LFS patients and establish a theoretical foundation for targeting STIL as a potential molecular therapeutic target for OS.

## Methods

### Data collection and data processing

We downloaded microarray and scRNA-seq datasets related to LFS and OS from the GEO database (http://www.ncbi.nlm.nih.gov/geo). In total, we obtained one gene expression dataset for LFS, seven gene expression datasets for OS, and two scRNA-seq datasets for OS. To ensure data consistency and preprocessing of all data, we applied the ComBat algorithm from the “SVA” package to correct for batch effects in the non–scRNA OS datasets and obtained clinical information^49^. Detailed information of all datasets, including microarray platforms, and sample information, are shown in Table 1.

**Table 1:** Summary of GEO datasets included in this study

ID	Dataset	Platform	Disease	Sample source	Control samples	Disease sample	Application
1	GSE23994	GPL570	LFS	Epithelial cells	8	16	Screening WGCNA of LFS
2	GSE19276	GPL6848	OS	Primary tumor	5	23	Screening DEGs of OS
3	GSE28424	GPL13376	OS	Cell lines	4	19	Screening DEGs of OS
4	GSE33383	GPL10295	OS	Primary tumor	15	84	Screening DEGs of OS
5	GSE36001	GPL6102	OS	Cell lines	6	19	Screening DEGs of OS
6	GSE42572	GPL13376	OS	Mesenchymal stromal cells	5	7	Screening DEGs of OS
7	GSE16091	GPL96	OS	Primary tumor	NA	34	Acquiring Clinical Information
8	GSE21257	GPL10295	OS	Primary tumor	NA	52	Acquiring Clinical Information
9	GSE162454	GPL24676	OS	Primary tumor	NA	6	scRNA-seq analysis
10	GSE152048	GPL24676	OS	Primary tumor	NA	11	scRNA-seq analysis

### Correlation module analysis of LFS

LFS, as a hereditary cancer susceptibility syndrome driven by germline TP53 mutations, may induce systemic network-level perturbations. Therefore, to capture these complex co-expression patterns, we employed WGCNA to identify gene modules associated with the LFS phenotype^50^. We performed log2 transformation and normalization to the expression matrix, and filtered out genes with a standard deviation below 0.5. After excluding the abnormal samples using the “goodSamplesGenes” function, we selected the optimal soft-thresholding power with the “pickSoftThreshold” function to construct an adjacency matrix, followed by calculation of the topological overlap matrix (TOM) and the gene ratio and the corresponding dissimilarity (1–TOM). Modules were identified using the dynamic tree cut method, with a minimum module size set to 50 genes and the module eigengene dissimilarity threshold set to 0.25. Upon identifying the LFS-associated module, we opted to include all module genes in the initial step of our downstream analysis rather than restricting the selection to hub genes. This inclusive strategy was chosen to create a comprehensive candidate pool, ensuring that biologically significant genes that are not topologically central to the network were not prematurely excluded.

### Screening of differentially expressed genes in OS

For OS, we primarily aimed to identify genes exhibiting significant and robust expression changes in tumor tissues compared to normal controls. To achieve this, we merged five OS datasets using the limma package and performed differential expression analysis to identify disease-associated DEGs. For datasets containing technical duplicates, the duplicateCorrelation method in limma is used to estimate the correlation between technical duplicates. The Benjamini–Hochberg method was applied, with adjusted *p* < 0.05 and |logFC| > 0.585 set as the threshold for DEGs identification. The “ggplot2” and “pheatmap” packages were used to visualize DEGs in OS. The VennDiagram package was employed to compare LFS-associated module genes with OS DEGs, aiming to identify LFS-OS shared genes, thereby exploring the potential mechanisms underlying the predisposition of LFS to OS^51^.

### Functional enrichment analysis

To reveal the potential pathogenic mechanisms linking LFS and OS, we performed GO and KEGG enrichment analyzes on the shared genes using the “clusterProfiler” package, with a significance threshold of *p* < 0.05^52^. GO enrichment analysis was used to identify functional categories of genes, including BP, CC, and MF, while KEGG analysis focused on identifying key signaling pathways. Functional clustering and dimensionality reduction of GO and KEGG were performed using the “enrichplot” and “ggplot2” package to demonstrate the functional similarity and clustering relationships among enriched terms.

### Feature genes selection using machine learning

To further identify robust and consistent feature genes for LFS-OS, we employed three machine learning algorithms for feature selection: RF, LASSO, and SVM-RFE, to identify feature genes. Although this conservative consensus strategy carries the risk of omitting genes not jointly identified by all three methods, it maximizes reliability and robustness. First, LASSO logistic regression was performed using the “glmnet” package, with the optimal lambda value determined by tenfold cross-validation. Candidate feature genes were selected based on the lambda.1se criterion, which corresponds to the largest lambda within one standard error of the minimum mean squared error^53^. Next, the “e1071” package was used to implement SVM-RFE. Candidate biomarkers were ranked by recursive feature elimination, and the performance of each feature subset was evaluated via tenfold cross-validation error, with top-ranking genes retained for further analysis^54^. Finally, the RF algorithm was applied using the “randomForest” package, and genes with MeanDecreaseGini index greater than 1.0 were selected as candidate feature genes^55^. The intersection of candidate feature genes derived from the three machine learning algorithms was then taken to obtain robust and consistent feature genes.

### Evaluating the classification performance of feature genes

To evaluate the classification performance of the selected feature genes, we employed a merged and normalized expression matrix from 5 OS datasets, comprising a total of 187 samples (35 controls and 152 OS cases). The dataset was randomly divided into a training set (80%) and a test set (20%) using stratified sampling to maintain the case-control ratio. This resulted in a training set comprising 150 samples and a test set comprising 37 samples. To optimize each model, we employed the automatic hyperparameter tuning capabilities within the caret package to evaluate ten widely used machine learning algorithms. During 20-fold cross-validation, caret tests the default algorithm-specific parameter tuning grid^56^. The set of hyperparameters that yielded the highest average AUC across the cross-validation folds was automatically selected to train the final model. Interpreting the best model using SHAP and visualizing it to visualize the importance and interactions of the features allows us to have a more comprehensive understanding of the extent to which the feature genes contribute to the classification results^57^.

### Construction and validation of the nomogram for clinical application

To develop a clinical prediction tool based on feature genes, we constructed a logistic regression model for the target variables. A multivariable logistic regression model was fitted using the rms package to integrate the feature genes, and a nomogram was subsequently constructed to visualize individualized risk predictions^58^. In the nomogram, the relative expression level of each feature gene corresponds to an individual score, and the total score is calculated by summing the points for all variables, which is then used to estimate the personalized disease risk. To validate the robustness of the model, fivefold cross-validation was performed using the “caret” package to evaluate the model’s discrimination and stability. Finally, the model was refitted on the entire dataset to generate the nomogram, calibration curve, DCA, and clinical impact curve.

### Clinical and immune relevance of feature genes

To verify the clinical significance and potential biological function of the feature genes in OS, we performed a comprehensive multidimensional analysis, including metastasis association analysis, KM survival analysis, GSEA, and immune cell infiltration analysis. First, we introduced an external validation dataset (GSE21257) and used the Wilcoxon rank-sum test to assess differences in feature genes expression between metastatic and non-metastatic OS samples. Subsequently, we used the “survival” and “survminer” packages to plot KM survival curves for overall survival in the external datasets (GSE21257 and GSE16091). Survival time was right-cutoff at 5 years, and patients were stratified into high- and low-expression groups according to the mean expression level of each feature gene. Log-rank tests were used to assess the statistical significance of survival differences between groups, in order to determine the prognostic impact of differential feature genes expression in OS patients.

Pathway enrichment analysis was performed using the GSEA function in the “clusterProfiler” package, with the KEGG pathway gene set (c2.cp.kegg) as the reference and a significance threshold of *p* < 0.05^52^. Based on the direction of the normalized enrichment score (NES), pathways significantly enriched in the high-expression and low-expression groups were extracted, and enrichment plots were generated for the top five significant pathways in each group. It was used to reveal the relationship between different expression levels of feature genes and pathway regulation.

Furthermore, to explore the potential relationship between feature genes and the immune microenvironment of OS, we performed immune cell infiltration analysis using the CIBERSORT algorithm^59^. The LM22 signature matrix was used as a reference, and 1000 permutations were conducted. Samples with *p* < 0.05 were considered high-confidence and included in subsequent analyzes. The Spearman correlation test was used to construct the correlation matrix between the feature genes and immune cells to assess the relationship between the expression levels of the feature genes and specific immune cell subpopulations. Meanwhile, we analyzed the expression profiles of 47 immune checkpoint genes (ICGs) based on STIL average expression levels. After excluding genes with minimal variation (SD < 0.001), we employed the Wilcoxon rank-sum test to compare ICGs expression differences between groups.

### Single-cell data sources analysis

This study included two scRNA-seq datasets from the GEO database: GSE162454 (6 samples) and GSE152048 (11 samples), with a total of 17 OS samples, and the data processing was performed using Seurat (v5.2.1)^60^. The initial filtering criteria were set as min.cells = 3 and min.features = 200. Quality control thresholds were defined as follows: nFeature_RNA between 300 and 5000, nCount_RNA > 1000, removal of the top 3% of cells with the highest UMI counts, mitochondrial gene percentage (mt%) < 10%, and hemoglobin gene percentage (HB%) < 3%.

The data were normalized using the NormalizeData function to select the first 3000 highly variable genes using FindVariableFeatures and standardization using ScaleData. After PCA was conducted, and batch effect correction was performed using Harmony (v1.2.1), with the top 30 principal components retained for downstream analyzes. For UMAP dimensionality reduction, the dims parameter was set to range from 10 to 30 (step size = 5), with a resolution of 0.2, and the optimal dims value was determined to be 10. The “clustree” package (v0.5.1) was used to evaluate clustering stability across resolutions from 0.1 to 1 (step size = 0.1), and a resolution of 0.2 was ultimately selected. Clustering was performed using FindNeighbors and FindClusters with dims = 1:10 and resolution = 0.2, resulting in the identification of 13 distinct cell subpopulations (Cluster 0–12), which were subsequently visualized.

### GSVA analysis at the scRNA-seq level

To assess the differences in functional status of various cell types, we applied the GSVA method (v2.0.7) to score the average expression profiles of each cell type within the Seurat object. The average RNA expression levels by cell type were calculated using the AggregateExpression function, and genes with non-zero expression were retained. The top 1000 highly variable genes with the largest standard deviations were further selected to construct a gene–gene correlation heatmap. The pathway annotations were derived from the Hallmark gene sets in the “msigdbr” package, and after constructing a GeneSetCollection object, the ssGSEA enrichment analysis was performed using the ssgseaParam and gsva functions. The results were subsequently visualized to display pathway activity across cell types.

### Developmental trajectory and cell stemness analysis

To evaluate the stemness status of malignant cells, CytoTRACE2 (v1.1.0) was applied to score stemness based on scRNA-seq data, where higher scores indicate lower differentiation and higher stemness properties. Subsequently, Monocle3 (v1.3.7) was used to construct developmental trajectories for three malignant cell subtypes—Pro-OSCs, OB-OSCs, and MG-OSCs—to uncover their potential evolutionary relationships. The inferred trajectories were embedded into the UMAP space and annotated by cell type to visualize the transition from precursor states to differentiated subtypes. Monocle3 automatically inferred the trajectory root and generated UMAP plots to display the dynamic gene expression changes along the differentiation paths.

### Gene expression dynamics and pseudotime analysis

To characterize the dynamic expression patterns of genes during the developmental process, pseudotime values were extracted for each cell. After removing NA/Inf values, cells were divided into five equal-width bins based on their pseudotime values. The expression matrix was transposed so that each row represented a cell, and pseudotime bin annotations were added. The data were then converted to long format using pivot_longer, and the average expression level of each gene was calculated within each bin (only genes expressed in cells with expression values ≥ 1 were retained). Bar graphs, box plots, with scatter plots were plotted using ggplot2, and trend lines were superimposed to show changes in expression. The Wilcoxon rank-sum test was applied to assess differences between adjacent bins.

### Copy number variation and cell communication analysis

To identify potential malignant cells, inferCNV (v1.22.0) was used to perform copy number variation (CNV) analysis on the single-cell dataset. Parameters were set as cutoff = 0.1, denoise = TRUE, and cluster_by_groups = TRUE. CNV values were extracted and normalized, and a CNV score (cnvScore) was calculated for each cell to assess the level of genomic instability, thereby aiding in the identification of cell populations with malignant characteristics.

To systematically investigate the cell communication patterns between STIL-high Pro-OSCs and other cell types, the “CellChat” package (v1.6.1) was used to construct intercellular communication networks. The analysis was based on the normalized expression matrix and cell type annotations, with ligand–receptor information derived from CellChatDB.human. Highly expressed ligand–receptor pairs were further mapped onto the protein–protein interaction network to enhance biological interpretability. After excluding subpopulations with fewer than 10 cells, aggregated communication networks were constructed, and the regulatory roles of each cell type within the network were evaluated. Visualization of the communication network included the number and strength of interactions between cell types, signaling pathway activities, contributions of key ligand–receptor pairs, and the specific roles of individual signaling axes, thereby uncovering the functional roles of different cells in the communication network.

### Drug sensitivity and pan-cancer analysis of STIL

To evaluate the association between STIL expression levels and drug sensitivity, we retained only OS samples and divided them into high and low expression groups based on the mean STIL gene expression. Subsequently, drug response data (GDSC2_Res) and gene expression data (GDSC2_Expr) for all cell lines from the GDSC2 database (https://www.cancerrxgene.org/) were used as the training set, and the calcPhenotype function in the “oncoPredict” package was employed to predict the IC50 of various anticancer drugs in OS samples^61^. The Wilcoxon rank-sum test was then used to identify candidate drugs showing significant differences in predicted sensitivity between the high and low STIL expression groups (*p* < 0.001).

To evaluate the expression differences and prognostic value of the STIL gene in various cancer types, we performed a pan-cancer analysis by integrating gene expression and clinical phenotype data from The Cancer Genome Atlas (TCGA) and the Genotype-Tissue Expression project, covering 33 common human cancers. Two-sample t-tests were conducted to compare STIL expression between tumor and normal tissues within each cancer type, aiming to reveal its expression patterns and tissue specificity across different malignancies. Furthermore, Cox univariate analysis was performed on the TCGA pan-cancer cohort to assess the prognostic relevance of STIL expression in terms of overall survival, disease-specific survival, disease-free interval, and progression-free interval.

### Cell culture and transfection

SaOS-2, U-2 OS, and 143B cell lines were obtained from the Cell Bank of the Chinese Academy of Sciences (Shanghai, China). All cell lines were cultured in Gibco growth medium supplemented with 10% fetal bovine serum (FBS; Gibco) and 1% 100× Penicillin–Streptomycin (Gibco). The small interfering RNAs targeting STIL (genOFF h-STIL_1999A, SIGS0007865-1) and the negative control siRNA (siNC) were synthesized by RiboBio Co., Ltd (Guangdong, China). Using the riboFECT CP Transfection Kit (C10511-05, RiboBio), siSTIL was mixed with the kit (final concentration 50 nM) according to the manufacturer’s instructions and incubated at room temperature for 15 min to prepare the transfection complex. Cells were transfected when cell density reached 30%-40%. The knockdown efficiency of STIL was validated by western blot at 72 h post transfection.

### Western blot assay

Cells were lysed using RIPA lysis buffer (G2002, Servicebio, China). Total proteins were separated by 10% SDS-PAGE and transferred to methanol-activated PVDF membranes. After blocking with 5% non-fat milk, the membranes were incubated overnight at 4 °C with primary antibodies, including anti-p53 (GB12626, Servicebio, China), anti-STIL polyclonal antibody (PA5-87832, ThermoFisher, China). Subsequently, the corresponding HRP conjugated goat anti-mouse/rabbit IgG secondary antibody (GB23301/GB23303, Servicebio, China) was applied at room temperature for 1 h. Finally, protein bands were visualized using an enhanced chemiluminescence detection system.

### Colony formation assay

U-2OS and 143B cells were seeded into 6-well plates at a density of 1000 cells per well, with three biological replicates for each condition. The culture medium was refreshed every 3 days, and colonies were allowed to grow for 14 days. Cells were fixed with 10% paraformaldehyde for 10 min and subsequently stained with 0.1% crystal violet for 10 min to visualize colony formation.

### Wound-healing and tumor sphere formation assay

U-2OS and 143B cells were seeded into 6-well plates and grown to confluence. The straight lines were scratched with 10-μL pipette tips, and the wells were washed twice with PBS to remove detached cells. Wound closure was monitored at 0, 24, and 48 h to assess migratory capacity. U-2OS and 143B cells (4 × 10⁴ per well) were resuspended in sphere-forming medium supplemented with β-FGF, B27, and EGF (Proteintech, Wuhan, China), and plated onto ultra-low attachment 6-well plates. Then culture the cells at 37 °C and 5% CO₂ for 10 days. Count the number of spheroids (diameter >75 μm) under a microscope to assess sphere-forming efficiency.

### Statistical analysis

All data are presented as mean ± standard deviation (SD). For in vitro cell-based assays, each experiment included three replicates. Statistical differences between groups were analyzed using Student’s *t*-test or one-way ANOVA test. Overall survival estimates were calculated using Kaplan–Meier survival analysis. Wilcoxon rank-sum tests were used to compare gene differences among groups, drug sensitivity, ICGs expression variations, and pseudotime intervals. Statistical analysis for cell experiments was performed using GraphPad Prism software (version 8.0), while the bioinformatics section utilized R version 4.4.2. Statistical significance is indicated as follows: ^*^p < 0.05, ^**^p < 0.01, ^***^p < 0.001.

## Supplementary information

Supplementary-reviesed

## Data Availability

The raw data can be found in the GEO (https://www.ncbi.nlm.nih.gov/geo/) database. The raw data that support the findings of this study are available from the corresponding author upon reasonable request.

## Code availability

No new algorithms were developed for this article. All codes used in this analysis were run in R version 4.4.2 and are available from the corresponding authors upon reasonable request.
