# Supplementary material for: Multi-omics analysis reveals the key role of STIL in Li-Fraumeni syndrome and osteosarcoma
Source: NPJ Precis Oncol. 2026 Apr 20;10:159. doi: 10.1038/s41698-026-01432-y (PMC13096552; doi:10.1038/s41698-026-01432-y)

**Supplementary Table S1. Li-Fraumeni Syndrome and Osteosarcoma shared Genes**		
ADM	CKS2	MCM2
ANLN	CPA4	MCM4
APOD	CRIP2	NUSAP1
ASPM	ECHDC2	PBK
ATAD2	FADS3	RAD51AP1
AURKB	FXYD6	RNASEH2A
BUB1	FYCO1	RPA3
BUB1B	GAL	SOX18
C1RL	GMNN	SPATA18
CCNB2	H2AFZ	STIL
CDC20	IQGAP3	TOP2A
CDCA5	IRS2	TPX2
CDCA8	KIF20A	TRIM22
CDKN3	KIF2C	TRIP13
CENPA	KPNA2	TUBB2B
CENPF	LEPR	UBE2C
CEP55	MAD2L1	UBE2T

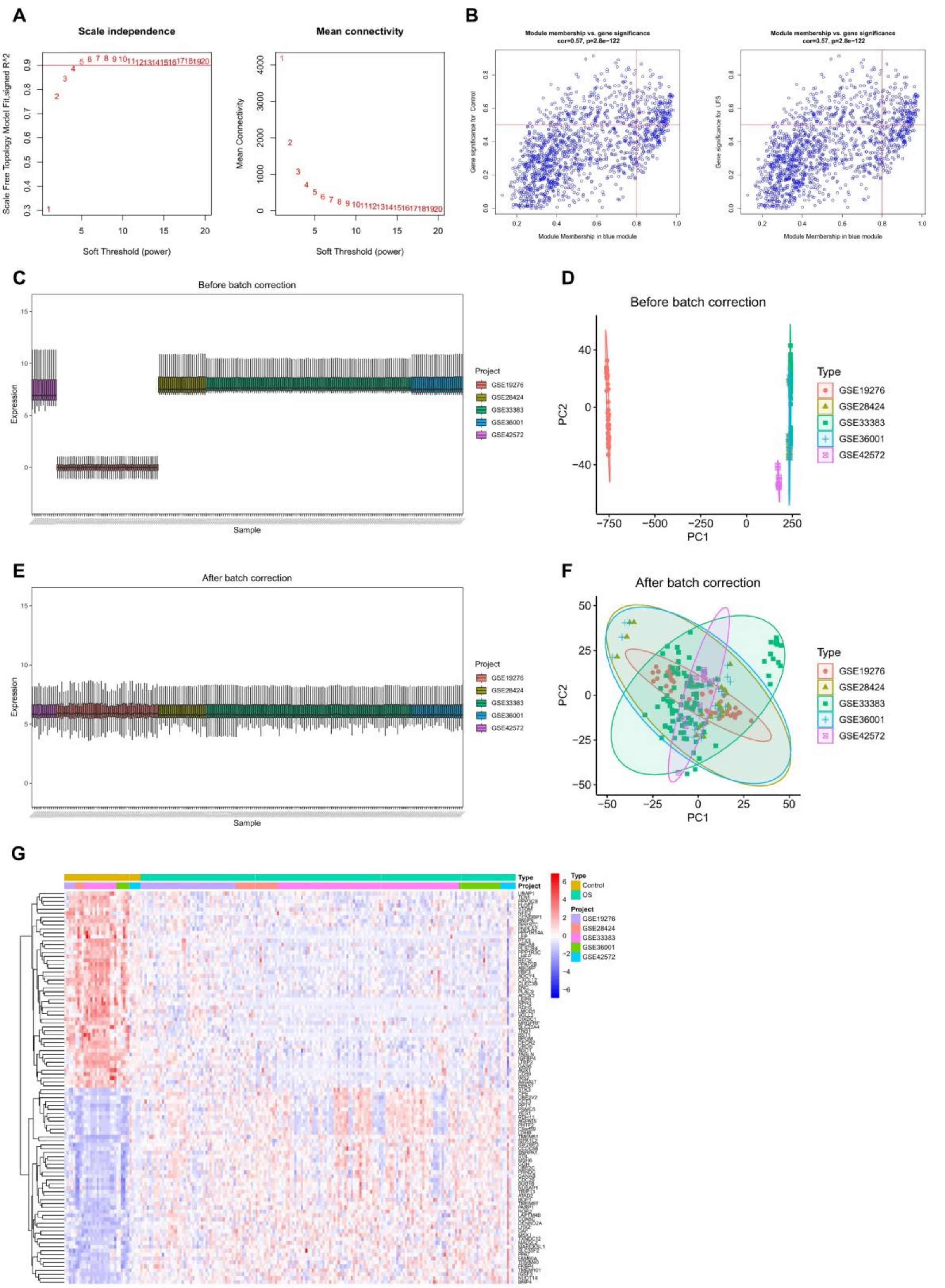

Supplementary Figure S1. Preprocessing of gene expression data for Li-Fraumeni Syndrome (LFS) and osteosarcoma (OS) shared gene acquisition.

(A) $\beta = 4$ was selected based on scale-free $R^2 \approx 0.9$ and mean connectivity. (B) Correlation between module membership and gene significance within the blue module (cor = 0.57, $p = 2.8 \times 10^{-122}$). (C) Boxplot of gene expression levels across samples before batch correction, showing visible variation across datasets (GSE19276, GSE28424, GSE33383, GSE36001, GSE42572). (D) PCA plot before batch correction reveals distinct clustering by dataset, indicating strong batch effects. (E) Boxplot of gene expression levels after batch correction using the ComBat algorithm, demonstrating improved expression distribution consistency across datasets. (F) PCA plot after batch correction shows reduced dataset-specific clustering and improved sample integration, suggesting effective removal of batch effects. (G) Heatmap of the top 50 up-regulated and top 50 down-regulated genes in OS.

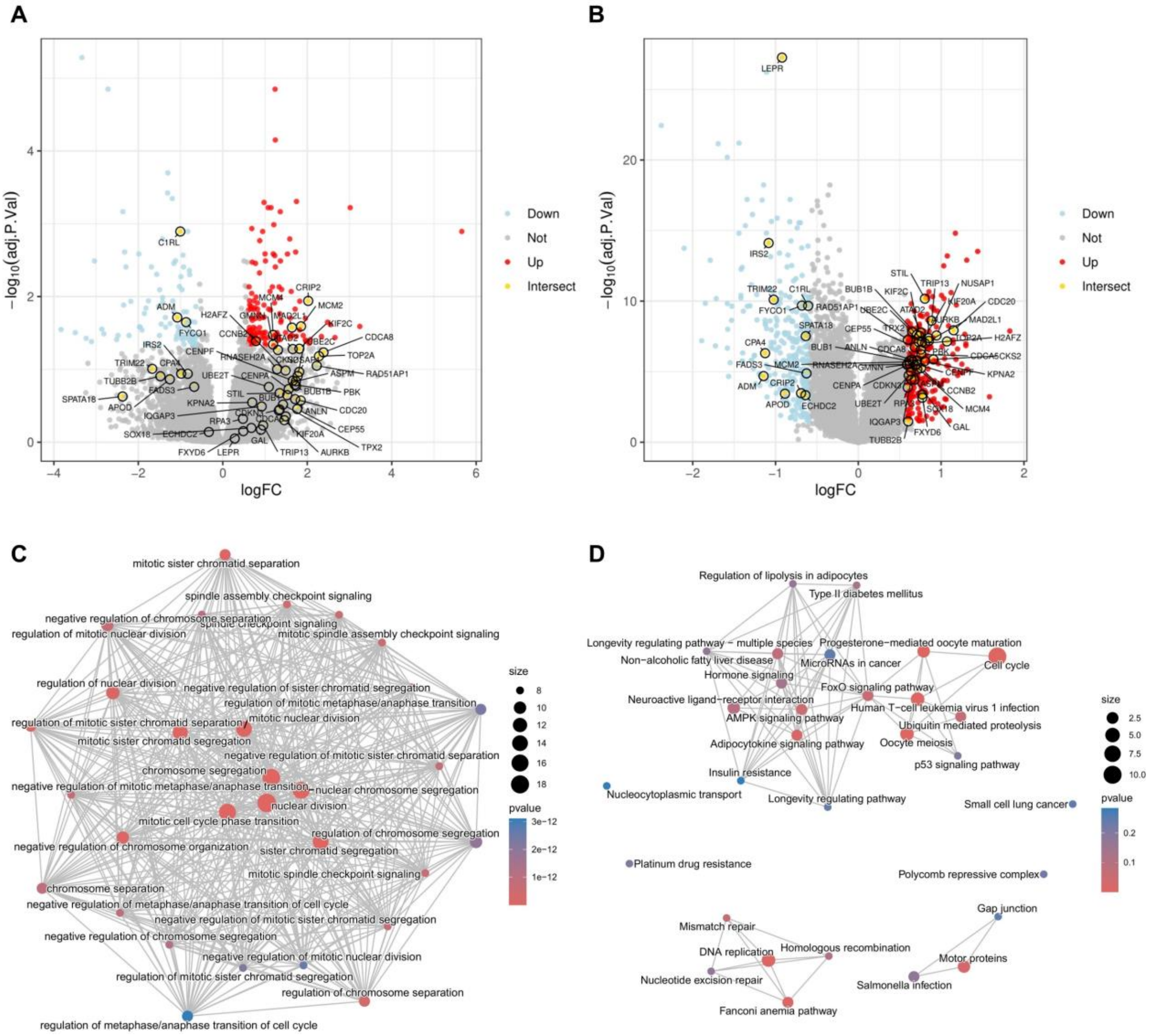

Supplementary Figure S2. Li-Fraumeni syndrome (LFS) and osteosarcoma (OS) shared gene expression status and functional enrichment analysis for dimension reduction.

(A) Expression status of 51 shared genes in LFS. (B) Expression status of 51 shared genes in OS. (C) GO items dimensionality reduction preprocessing. (D) KEGG items dimensionality reduction preprocessing. Node size indicates the number of genes and color indicates statistical significance.

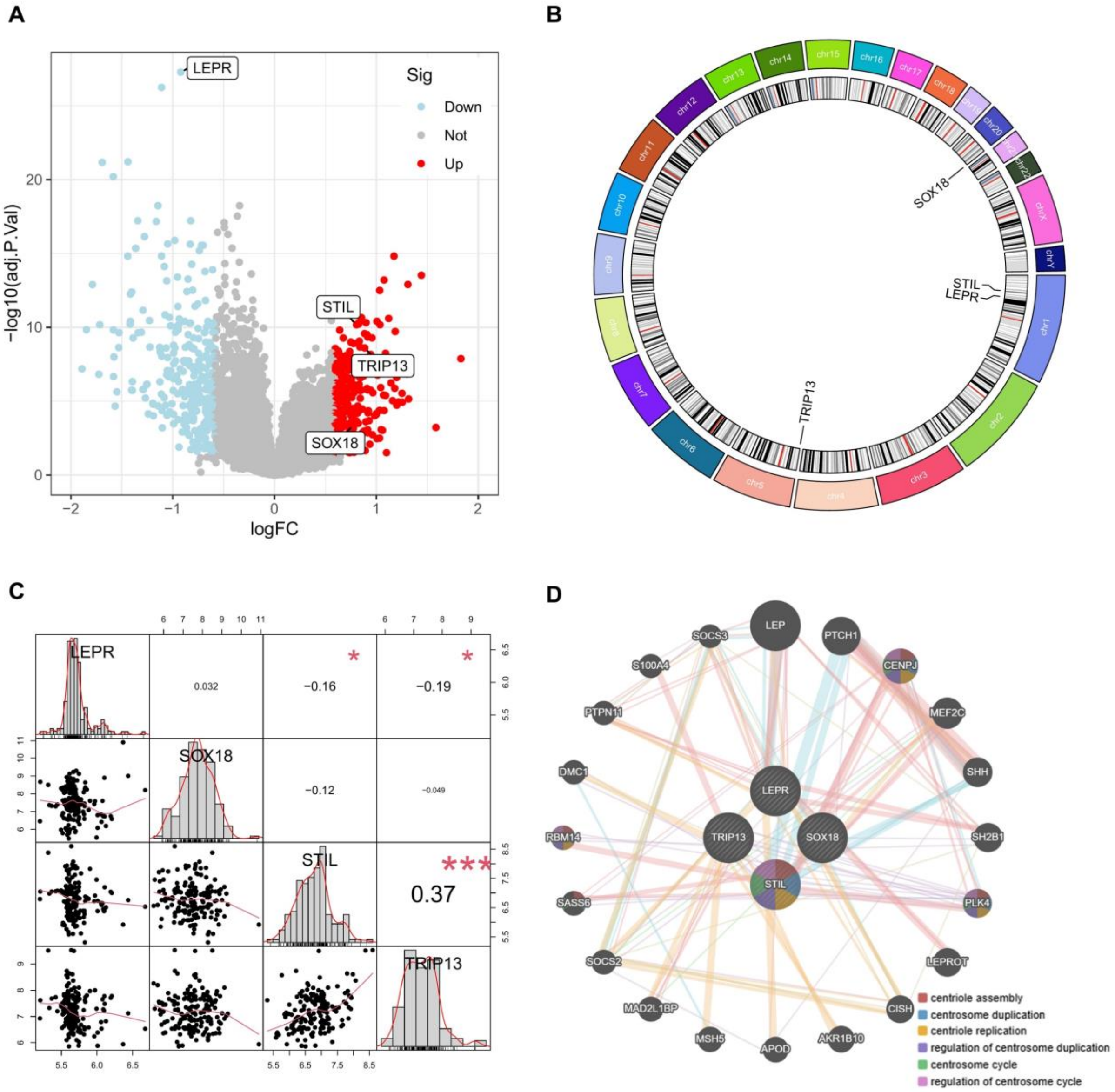

Supplementary Figure S3. Characterization of four feature genes.

(A) Volcano maps of feature genes. (B) Chromosomal localization of four feature genes. (C) Correlation analysis between the characterized genes: STIL was positively correlated with TRIP13, while LEPR was negatively correlated with both STIL and TRIP13. (D) Interaction network of the biological functions of the four feature genes. Different colors represent different biological functions. (**p*<0.05, ***p*<0.01, ****p*<0.001)

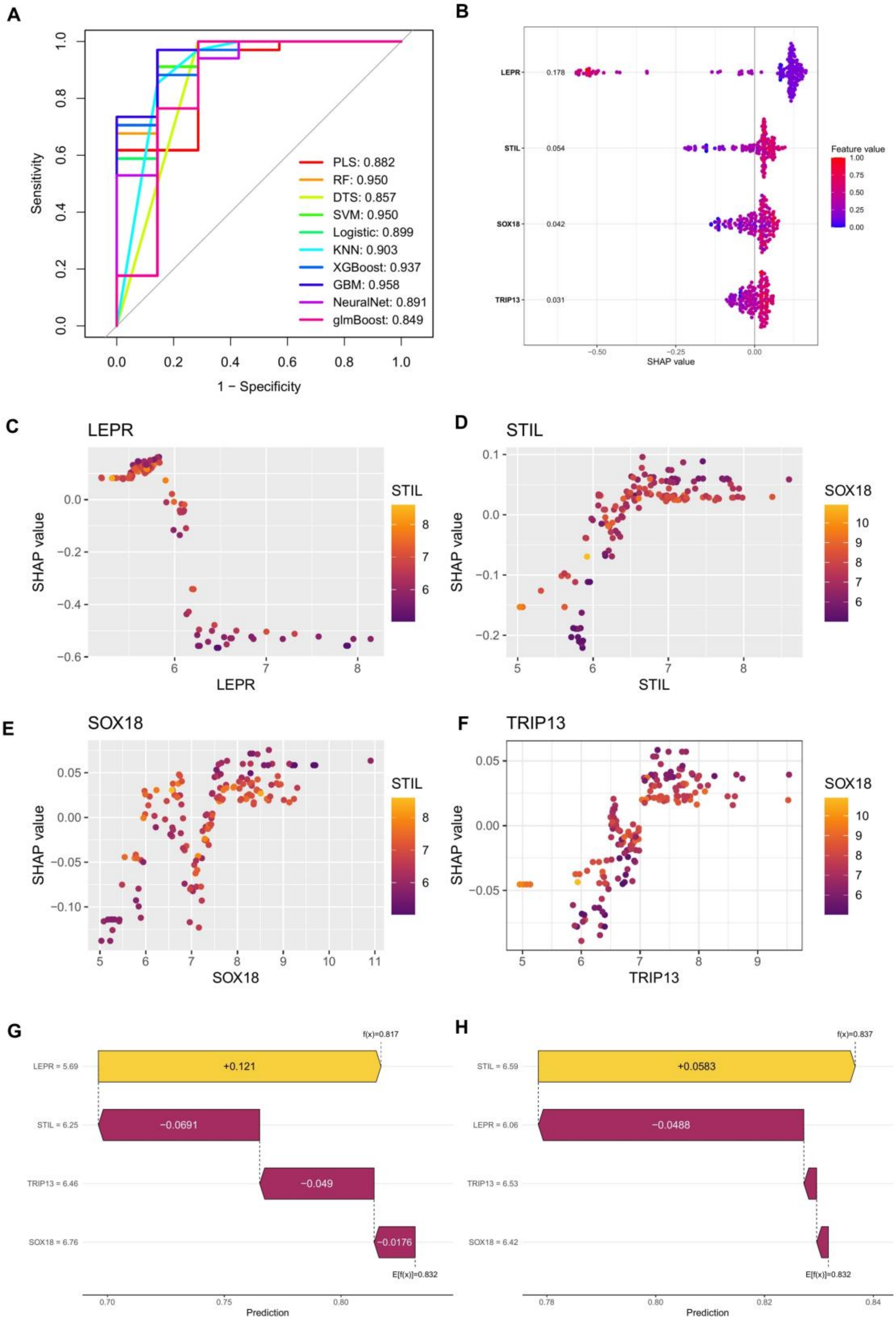

Supplementary Figure S4 Identification of optimal machine learning models and SHAP-based interpretability analysis of feature genes.

(A) ROC curves of ten machine learning models evaluating the classification performance of the four feature genes, GBM is the optimal model (AUC=0.958). (B) SHAP bee plot. The distributions of SHAP values for the feature genes and the impact on the predictions are shown. (C–F) SHAP dependence plots showing the relationship between gene expression levels and SHAP values for LEPR (C), STIL (D), SOX18 (E), and TRIP13 (F). Color gradients represent the expression levels of interacting genes, highlighting interaction effects between features. (G, H) SHAP waterfall plots E and F show the contribution of each characterized gene to the model prediction in normal sample GSM481115 and OS sample GSM481075, respectively. The yellow and rosy bars indicate positive and negative contributions to the forecast, respectively.

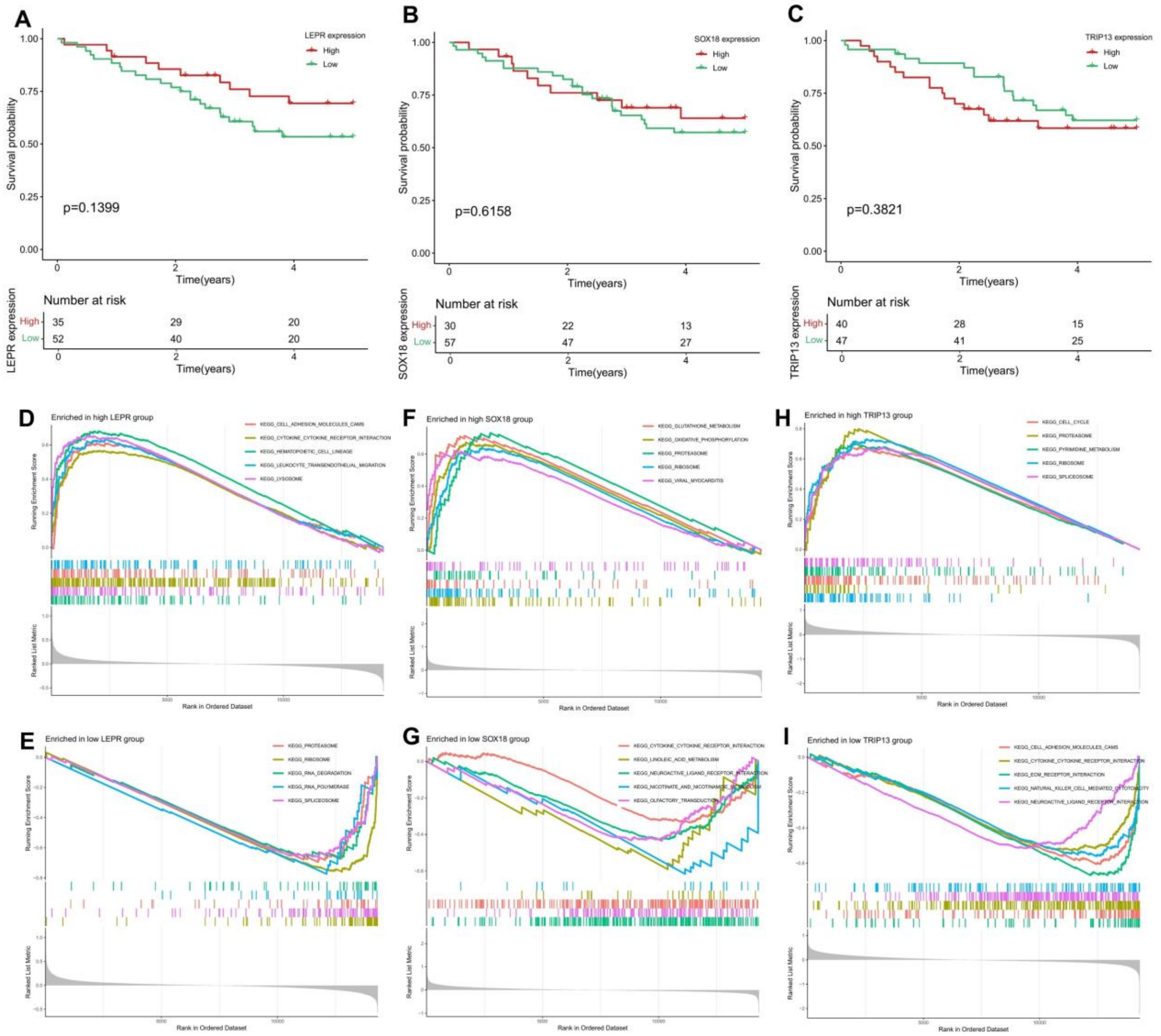

Supplementary Figure S5. Kaplan-Meier survival curves for and gene set enrichment analysis (GSEA) features genes.

Kaplan-Meier survival curves of OS patients with high and low expression of LEPR (A), SOX18 (B), and TRIP13 (C). GSEA pathways enriched results in the LEPR high expression (D) and low expression (E). GSEA pathways enriched results in the SOX18 high expression (F) and low expression (G). GSEA pathways enriched results in the TRIP13 high expression (H) and low expression (I).

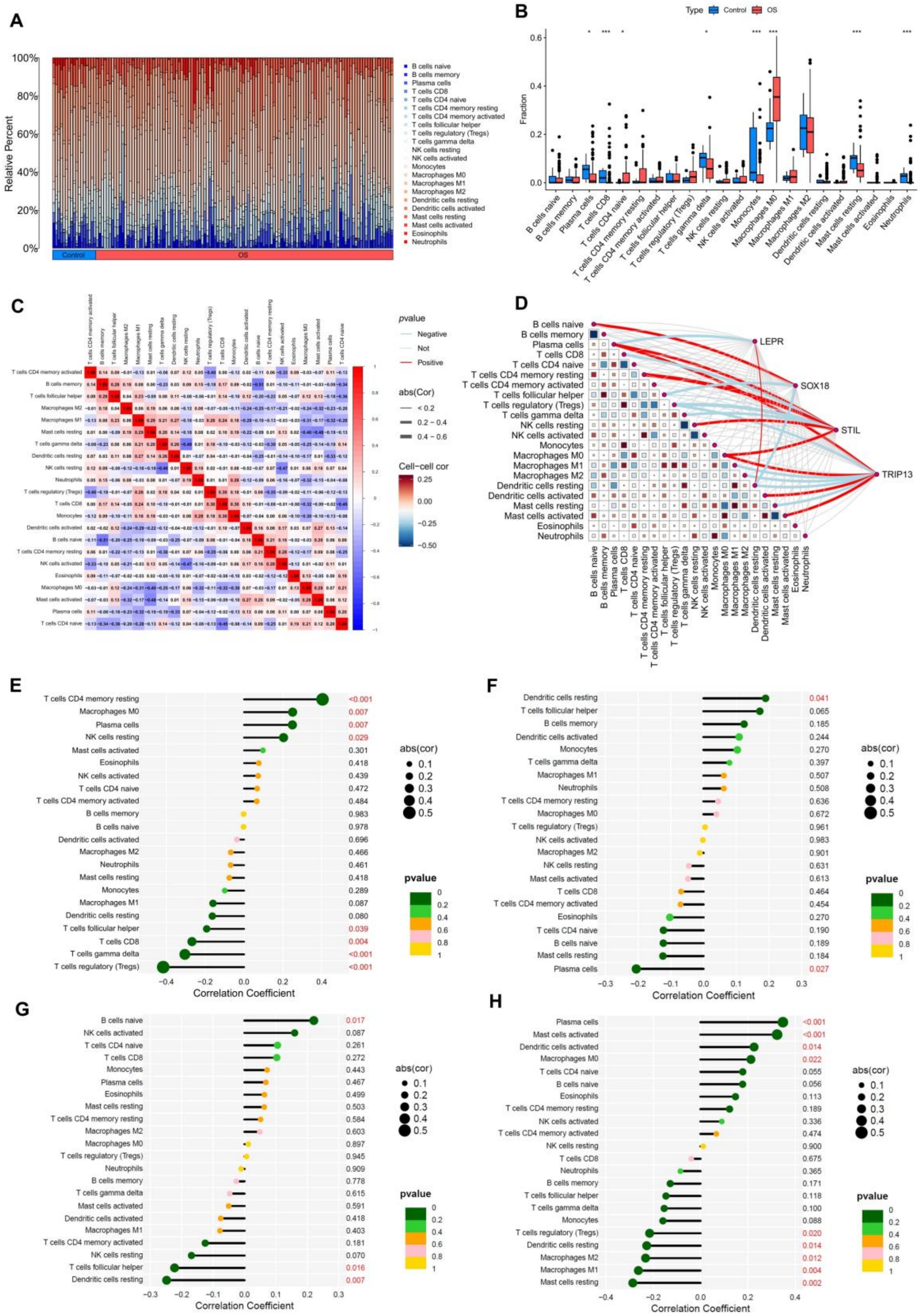

Supplementary Figure S6. Immune Infiltration Analysis in OS.

(A) Stacked bar graphs show the relative proportions of the 22 immune cell types in OS and normal control samples. (B) Box plots comparing the relative proportions of each immune cell type in the OS and control groups. (*$p < 0.05$, ** $p < 0.01$, *** $p < 0.001$). (C) Correlation heatmap illustrating the relationship between the 22 immune cell subpopulations in all samples. Red indicates positive correlation, blue indicates negative correlation. (D) Heatmap of the correlation between feature genes and immune cell infiltration levels. Red and blue lines indicate positive and negative correlations, respectively. (E-G) Lollipop plot illustrating the correlation coefficients between expression of STIL(E), LEPR (F), SOX18 (G), and TRIP13 (H) and 22 immune cell types.

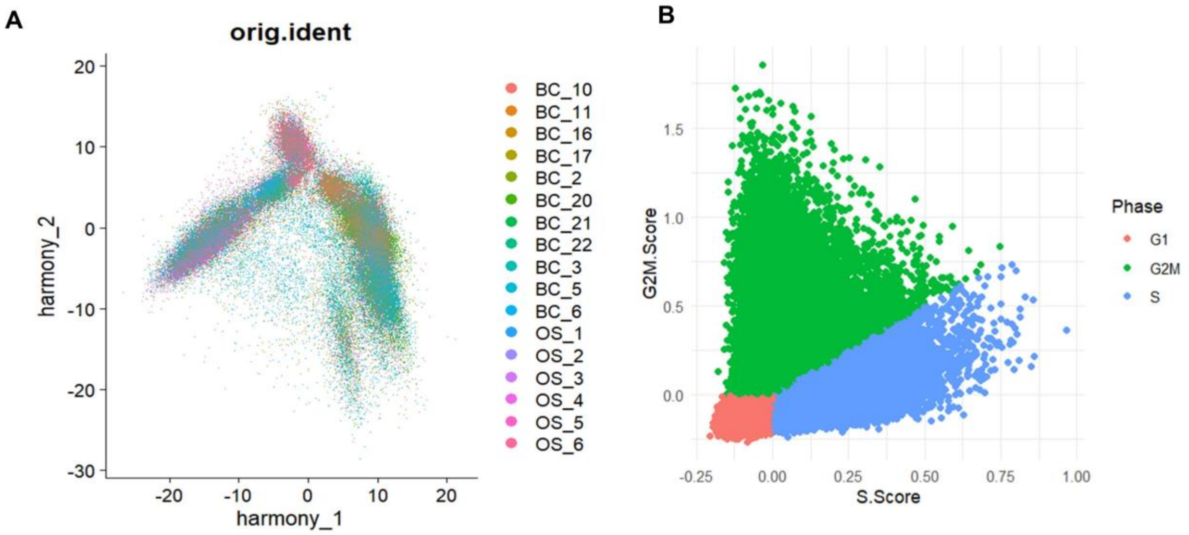

Supplementary Figure S7. Batch effect correction and cell cycle state analysis in single-cell transcriptomic data.

(A) UMAP plots before and after Harmony-based batch correction across datasets (GSE162454 and GSE152048). (B) Cell cycle phase scoring across all cells. Each dot represents a cell colored by its assigned phase (G1, S, G2/M).

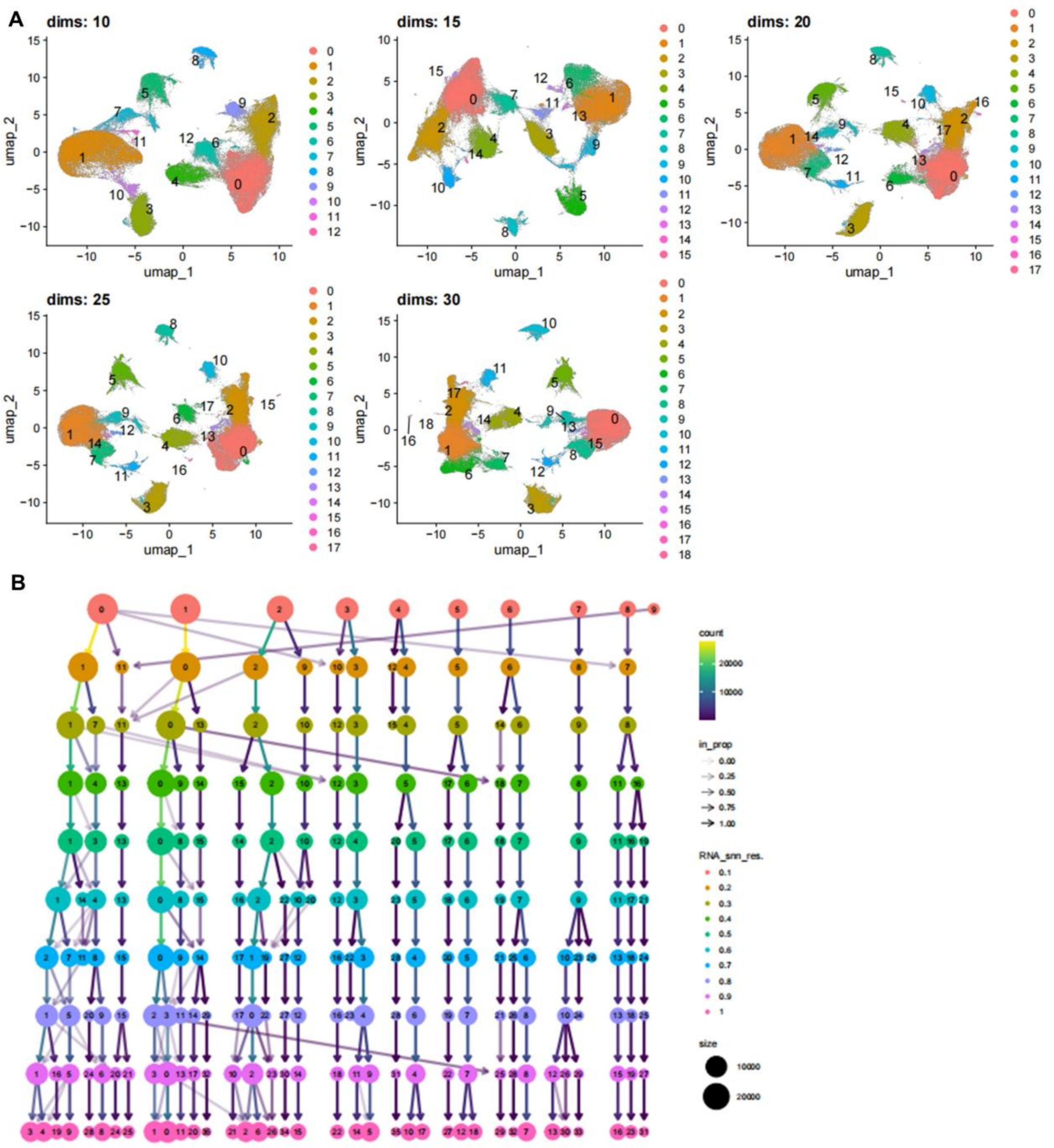

Supplementary Figure S8. Selection of dimensionality reduction parameter settings for osteosarcoma single cell data.

(A) UMAP plots showing the effect of different dimensionality parameters (dims = 10-30, step = 5) on clustering resolution for determining the optimal dims = 10. (B) Determination of resolution by clustree as 0.2

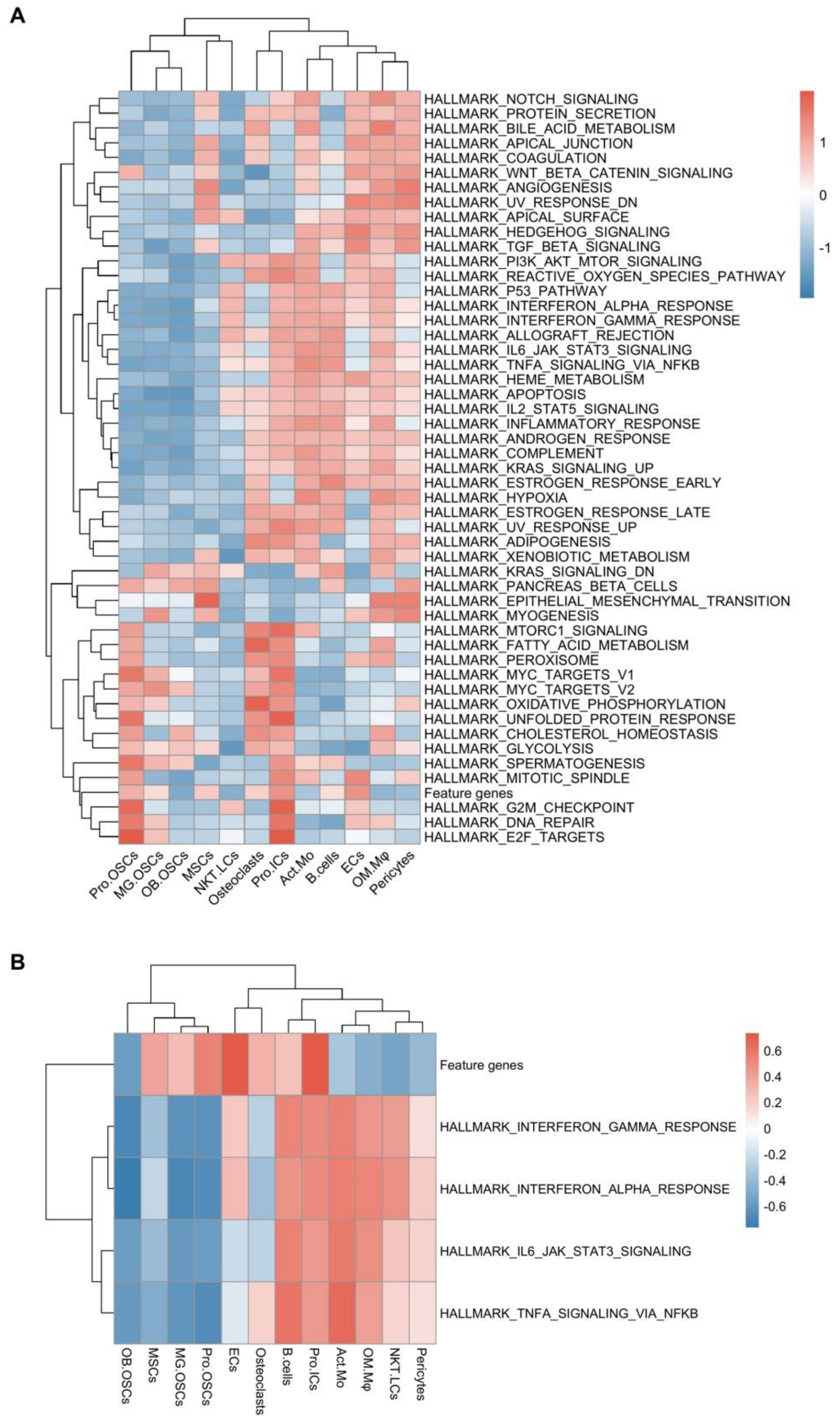

Supplementary Figure S9. GSVA pathway enrichment.

(A) Heatmap of GSVA pathway enrichment for Top 50 in different cell clusters. (B) Heatmap of GSVA pathway enrichment for Top 5 in different cell clusters.

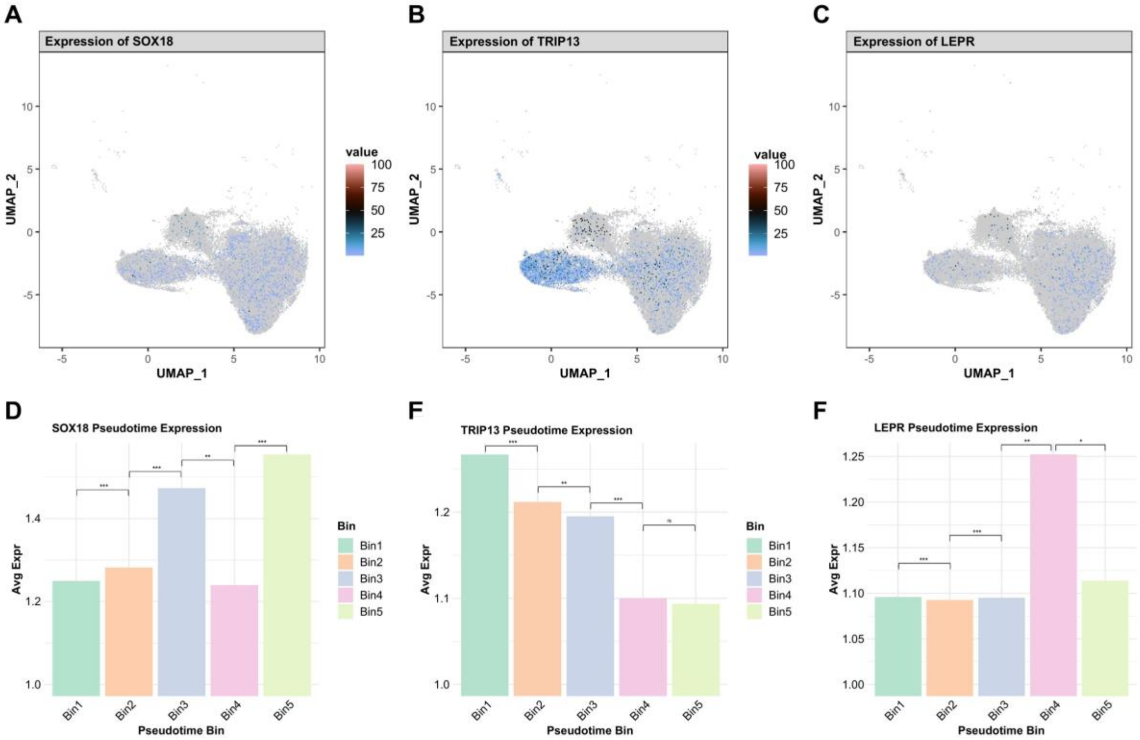

Supplementary Figure S10. Spatial distribution and pseudo-time analysis of feature genes.

(A-C) The UMAP plot of spatial features illustrates the expression patterns of the feature genes SOX18 (A), TRIP13 (B), and LEPR (C) within Pro-OSCs, OB-OSCs, and MG-OSCs. (D-F) Box plots of expression levels of feature genes SOX18 (D), TRIP13 (E), and LEPR (F) in 5 pseudotime bins.

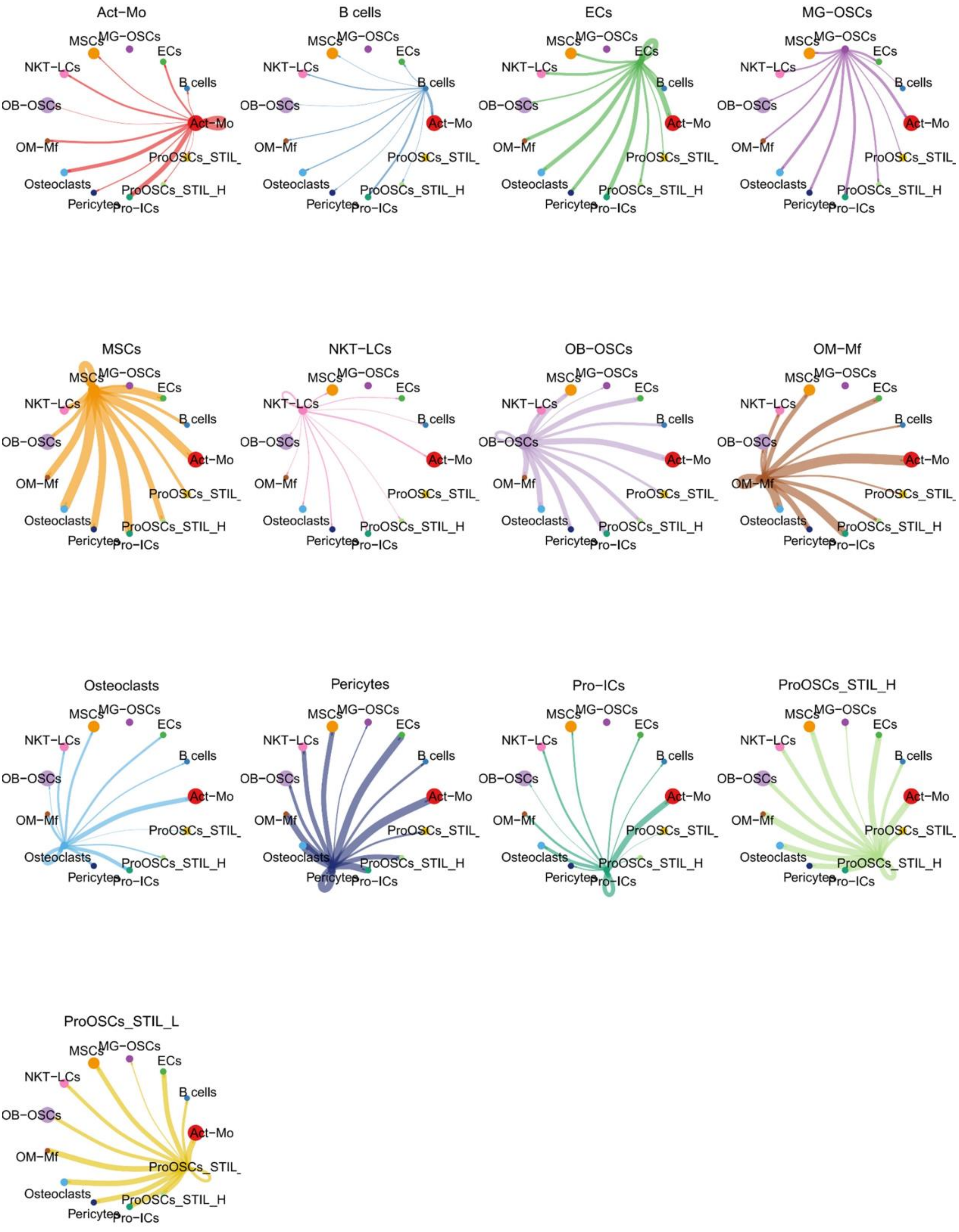

Supplementary Figure S11. Chord diagram showing outgoing interactions from sender clusters to Pro-OSCs-STIL-H and STIL-L subclusters.

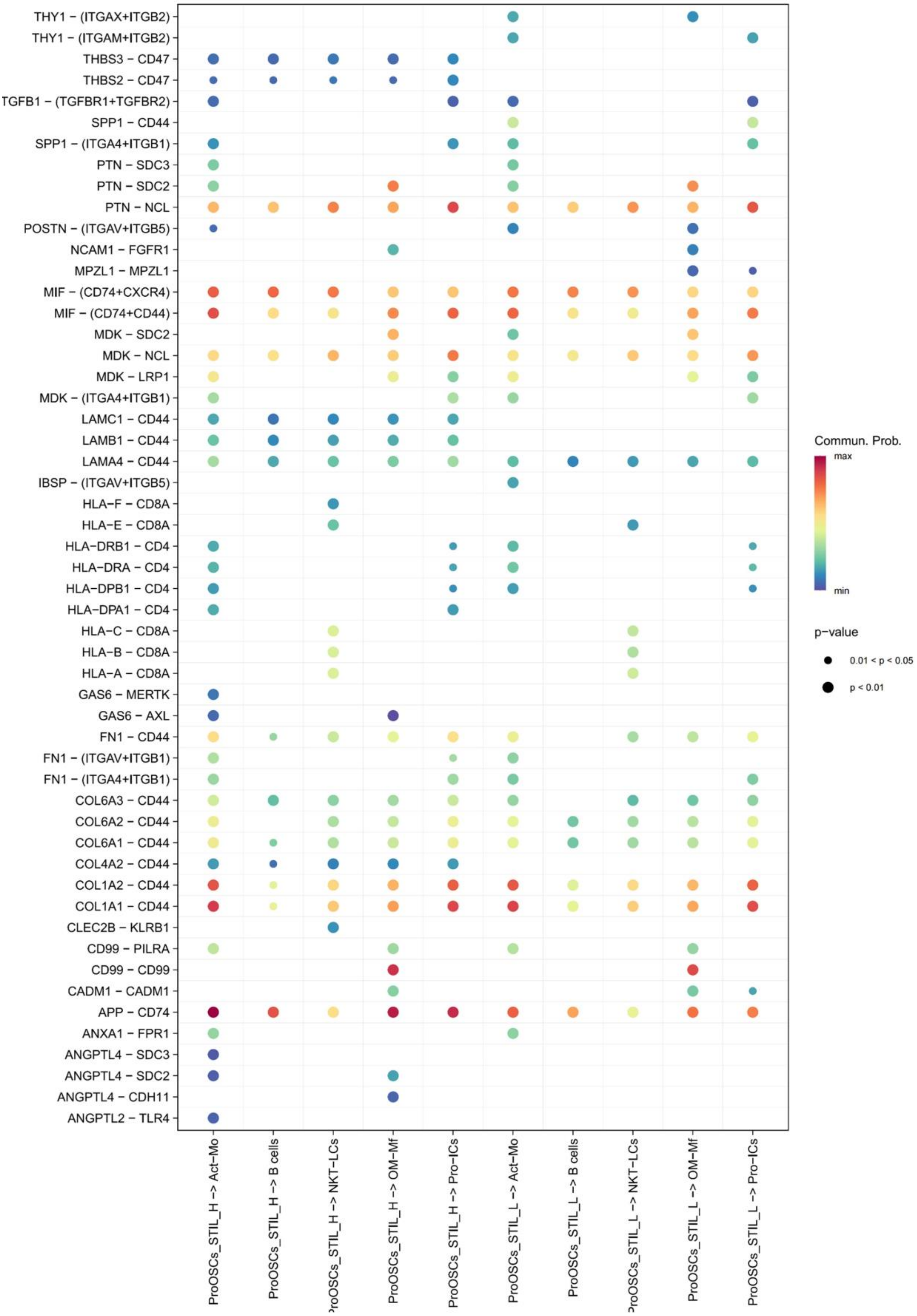

Supplementary Figure S12. Dot plots of differences in ligand-receptor interactions between Pro-OSCs-STIL-H and Pro-OSCs-STIL-L and immune-related clusters.

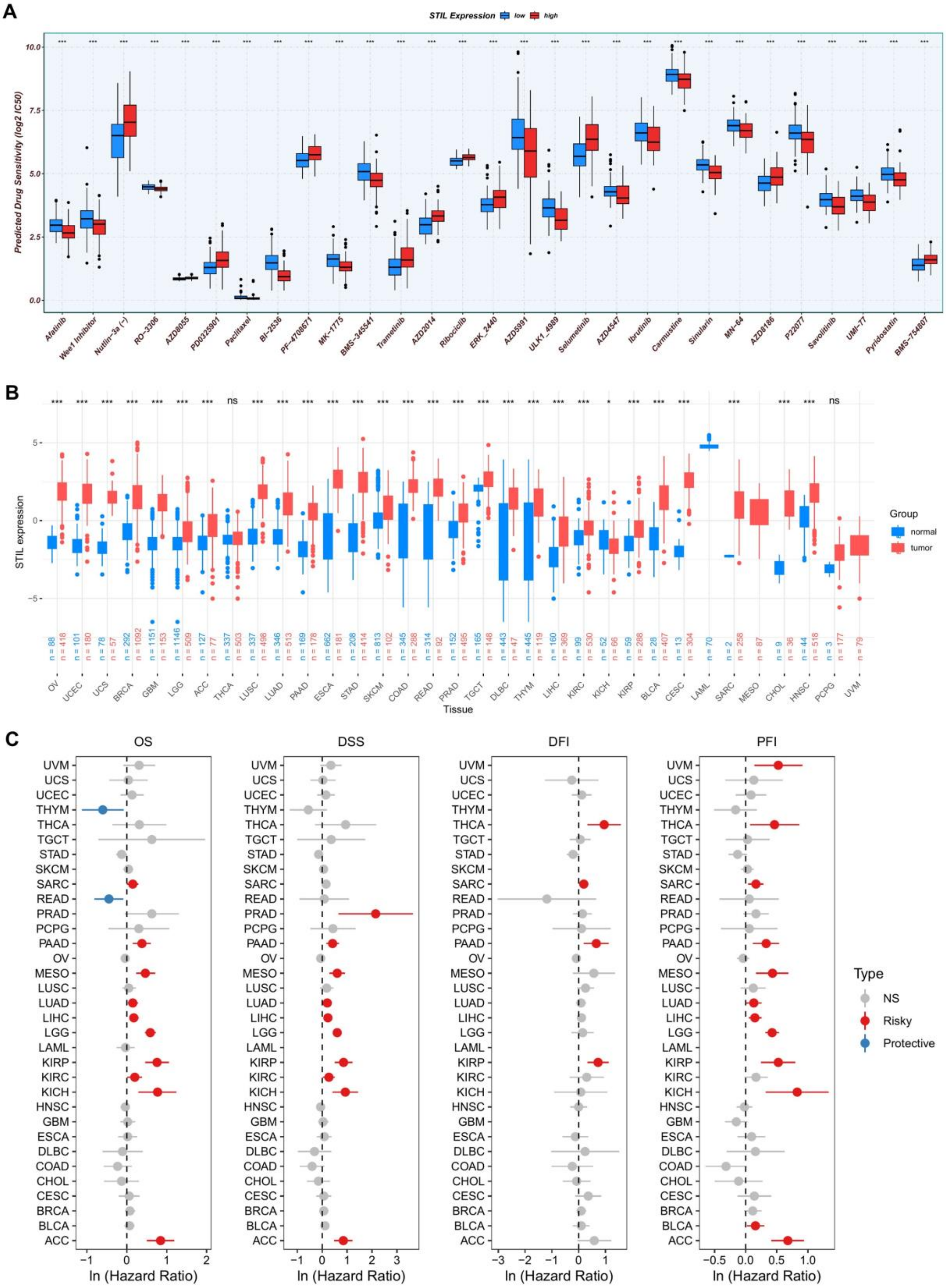

Supplementary Figure S13. Drug sensitivity and pan-cancer analysis of STIL expression.

(A) Drug sensitivity analysis for high- and low-expression of STIL. (B) Pan-cancer analysis of STIL expression. (*p < 0.05, **p < 0.01, ***p < 0.001, ns is not significant). (C) Forest plot of univariate Cox regression results of STIL expression in relation to overall survival (OS), disease-specific survival (DSS), disease-free interval (DFI), and progression-free interval (PFI) in different cancer types. Red and blue dots indicate that STIL is a risk factor and a protective factor.

Western blot raw figures

Figure 9A

STIL

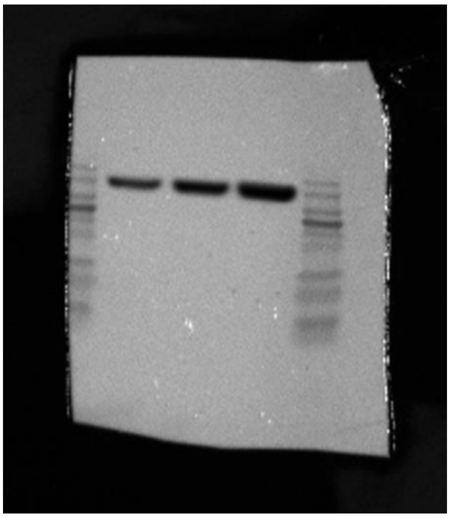

GAPDH

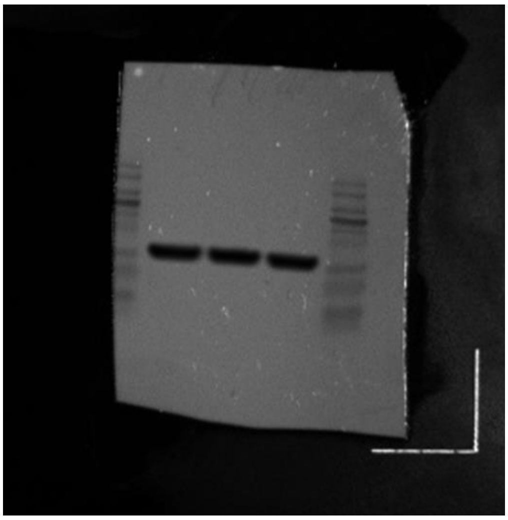

Figure 9B

STIL

U-2OS

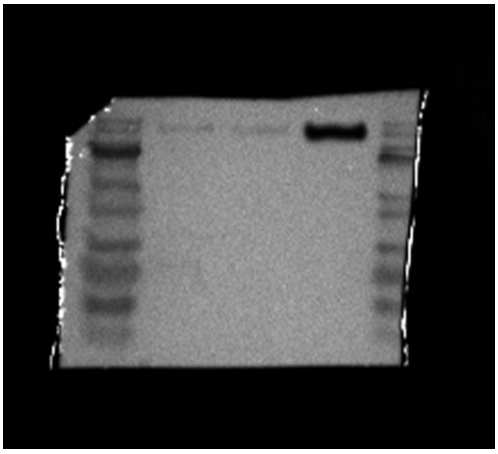

143B

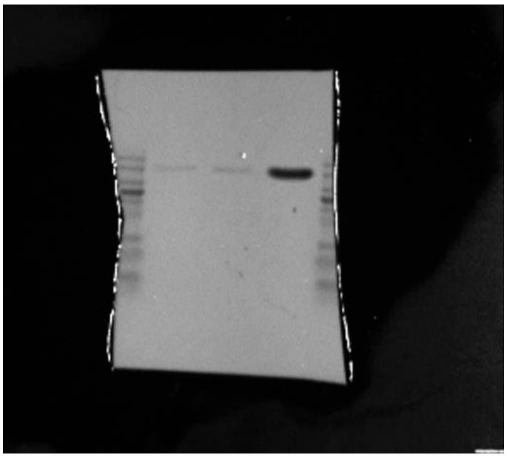

P53

U-2OS

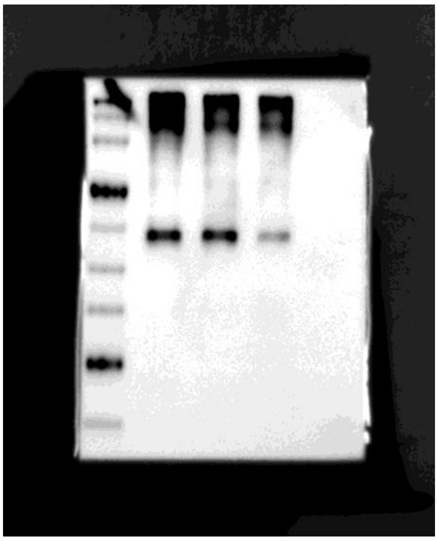

143B

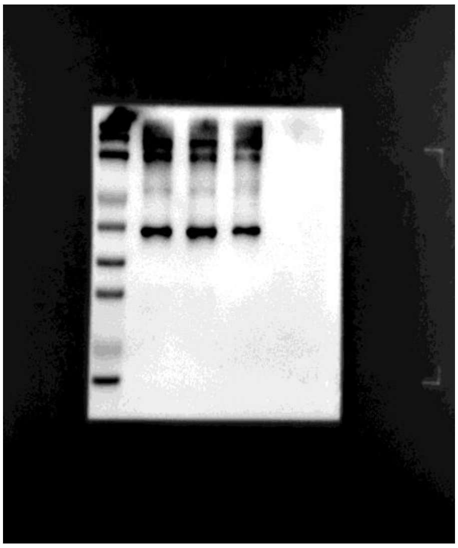

GAPDH

U-2OS

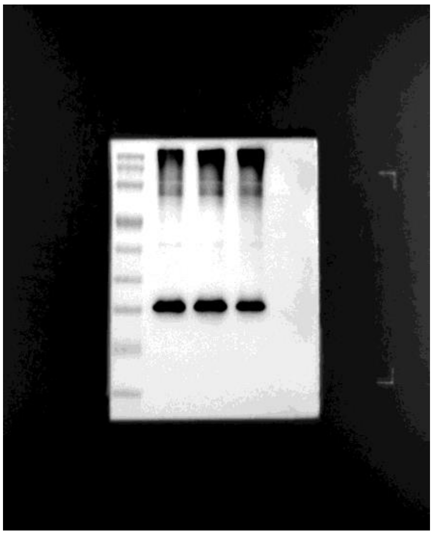

143B

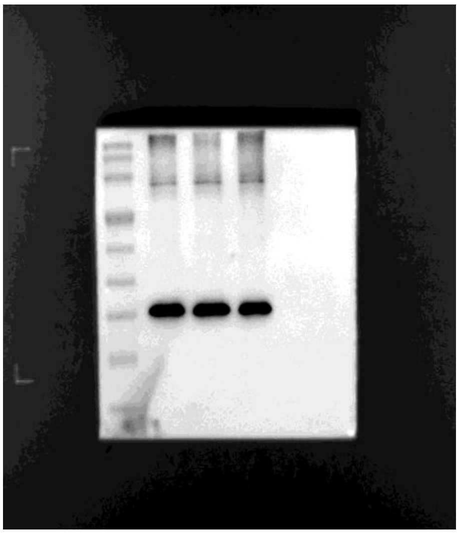

Supplement: Supplementary file 1 — Supplementary-reviesed [file 41698_2026_1432_MOESM1_ESM.pdf]
